# Genome-wide screening identifies ZFP91 as a key regulator of EVI1 in myeloid leukemia

**DOI:** 10.1038/s41388-026-03727-7

**Published:** 2026-04-25

**Authors:** Hiroki Hayashida, Yosuke Masamoto, Takashi Oyama, Toshiya Hino, Ken Morita, Katsunori Fujiki, Ryuichiro Nakato, Katsuhiko Shirahige, Mineo Kurokawa

**Affiliations:** 1 Department of Hematology and Oncology, Graduate School of Medicine, The University of Tokyo, Tokyo, Japan; 2 Department of Blood Transfusion, The University of Tokyo Hospital, Tokyo, Japan; 3 Department of Cell Therapy and Transplantation Medicine, The University of Tokyo Hospital, Tokyo, Japan; 4 Laboratory of Genome Structure and Function, Institute for Quantitative Biosciences, The University of Tokyo, Tokyo, Japan; 5 Laboratory of Computational Genomics, Institute for Quantitative Biosciences, The University of Tokyo, Tokyo, Japan; 6 Department of Cell and Molecular Biology, Karolinska Institutet, Stockholm, Sweden

**Keywords:** Acute myeloid leukaemia, Transcription

## Abstract

Ecotropic viral integration site 1 (EVI1) is essential for hematopoietic stem cell maintenance, and its aberrant expression is a significant adverse prognostic indicator in myeloid leukemia. EVI1 overexpression typically occurs due to chromosomal rearrangement involving 3q26. However, aberrant EVI1 expression is still observed in numerous cases without 3q26 abnormalities, leading to similarly poor outcomes, while the mechanism behind EVI1 overexpression in these cases remains largely unknown. Here, we performed genome-wide CRISPR screening using cells with GFP knock-in at the *EVI1* locus and identified zinc finger protein 91 (ZFP91) was the leading activator of EVI1. ZFP91 knockout significantly reduced EVI1 expression and cell proliferation. We also showed that ZFP91 binds to the *EVI1* promoter, enhancing H3K4me3/H3K27ac and chromatin accessibility. Our data showed that the ZFP91–EVI1 axis plays a critical role for activation of EVI1 in myeloid leukemia. Our screening approach represents a powerful and unbiased method for identifying expression regulators that can be broadly applied across a range of contexts.

## Introduction

Acute myeloid leukemia (AML), the most common form of acute leukemia in adults, is caused by a wide range of abnormalities in the transcriptional network. Among them, overexpression of stem cell-like transcriptional programs is an independent poor prognostic factor in AML [1]. However, in general, gene expressions are regulated by various mechanisms, including transcription factors that bind to promoters, transcription regulators that act on enhancers, and factors that alter chromatin structure, and it is not easy to elucidate the entire picture [2]. While recent advances in pooled genetic screening have enabled systematic interrogation of cellular pathways, studies that unbiasedly extract transcriptional regulators of a particular gene are still limited. Here, we demonstrate that a genome-wide clustered regularly interspaced short palindromic repeats (CRISPR) knockout screen can serve as an effective approach to identify novel regulators of *Ecotropic viral integration site 1 (EVI1)*, a strong prognostic factor in AML.

EVI1, a key player in maintaining hematopoietic stem cells [3, 4], is overexpressed in approximately 10% of AML patients, making it one of the strongest adverse prognostic factors for AML [5]. Aberrant EVI1 expression is thought to enhance the self-renewal ability of hematopoietic stem cells (HSCs) [6], inhibit hematopoietic differentiation [7], and induce myelodysplastic syndrome (MDS) [8] or AML [9] via various signaling pathways [9–12]. It is presumed to be closely related to the stem cell-like transcriptional program. However, because EVI1 is an extremely important transcription factor in maintaining normal hematopoiesis, it is difficult to directly target EVI1 in EVI1-overexpressing AML. Therefore, elucidating the mechanism of aberrant EVI1 expression is crucial for exploring targeted therapies of EVI1-associated AML.

Human EVI1 is encoded by the *MDS1 and EVI1 complex locus (MECOM)* gene on chromosome 3q26.2. AML with inv(3)(q21.3q26.2) or t(3;3)(q21.3;q26.2) is known to overexpress EVI1 through a well-known established mechanism in which the translocated *GATA binding protein 2 (GATA2)* distal enhancer drives EVI1 overexpression via the transcription factor MYB [13, 14]. Similarly, in AML with t(3;8)(q26;q24), the super-enhancer MYC is located to be near the *EVI1* promoter, leading to overexpression of EVI1. These mechanisms are called enhancer hijacking [15]. Other than this, specific translocations between 3q26 and other chromosomes can cause overexpression of EVI1 [16, 17]. Abnormal fusion proteins caused by chromosomal translocations involving *Lysine methyltransferase 2A (KMT2A)* at 11q23 bind to the *EVI1* promoter and activate its expression [18]. As shown above, chromosomal structural abnormalities play a major role in high EVI1 expression.

However, previous studies suggest that substantial proportion of EVI1-high AML is not associated with 3q26 abnormalities [19], although pericentric inv(3) aberration exists that cannot be detected by conventional chromosomal testing [20]. EVI1 overexpression has been reported to be a poor prognostic factor even in AML without 3q26 abnormalities, such as AML with normal karyotype [21]. The mechanism of EVI1 overexpression that is not mediated by chromosomal translocations also remains to be elucidated, but the whole picture is unclear.

To our knowledge, no comprehensive genome-wide exploration of the regulatory mechanisms of EVI1 expression has been reported. An analysis of transcription factor motifs in the sequence of the minimal promoter region of *EVI1* found that Runt-related transcription factor 1 (RUNX1) and ETS transcription factor ELK1 increase EVI1 expression [22]. Another report demonstrated that lymphoid enhancer-binding factor 1 (LEF1)/β-catenin complex binds to the *EVI1* promoter and enhances EVI1 expression in chronic myeloid leukemia (CML) [23]. A CRISPR knockout screen in AML with inv(3)(q21.3q26.2) using a custom library designed for the region between 3q21 and 3q26 has been reported [14], but the screen depends on motif databases. Limitation of these study designs is that only transcription factors with known motifs can be identified.

Therefore, in this study, we planned to perform a comprehensive genome-wide screening to investigate the mechanism of EVI1 overexpression in AML without chromosome 3q26 aberrations. Clarifying the regulatory mechanism of EVI1 expression and its differences between normal hematopoiesis and AML will contribute to a deeper understanding of the transcriptional network involving EVI1 and the discovery of therapeutic targets for EVI1-high AML.

Our screening successfully identified zinc finger protein 91 (ZFP91) as a novel lead activator of EVI1. *ZFP91* was first reported as an overexpressing gene in AML patients [24]. ZFP91 is an atypical E3 ubiquitin ligase [25], as well as a DNA-binding protein that functions as a transcription factor [26]. ZFP91 promotes cell growth and inhibits apoptosis in AML [27] and has been shown in previous studies to activate multiple oncogenic pathways, including nuclear factor-kappa B [25], mitogen-activated protein kinase [28], and hypoxia-inducible factor-1α [29]. However, there have been no reports on the function of ZFP91 in EVI1-associated AML.

## Methods

### Establishment of green fluorescent protein (GFP) knock-in cell lines

The single-stranded oligodeoxynucleotide repair template, prepared using Gibson assembly as previously described [14], was co-transfected into K562 cells with CRISPR-associated protein 9 (Cas9)/guide RNA (gRNA) ribonucleoprotein complex. After single-cell sorting, successful knock-in was confirmed by polymerase chain reaction (PCR) and Sanger sequencing.

### Pooled CRISPR screening

Human GeCKOv2 CRISPR knockout pooled library and Cas9-expressing lentiviral vector were provided from Feng Zhang (Addgene #1000000049) [30]. CRISPR screening was performed as previously reported [31].

### Quantitative reverse transcription PCR (qRT-PCR)

Reference genes for the initial qRT-PCR in K562, HEL, and CMK-11-5 cells were selected by GeNorm [32] and BestKeeper [33] algorithms. For the subsequent experiments, *PGK1* and *YWHAZ* were used as reference genes.

### ChIP experiments and ATAC-seq

Chromatin immunoprecipitation (ChIP) experiments and assay for transposase-accessible chromatin with high-throughput sequencing (ATAC-seq) library preparation were carried out as previously described [9, 34].

### Data analysis

Statistical analyses were mainly performed using GraphPad Prism 10 (RRID:SCR_002798). Detailed descriptions of the statistical analyses, including justification for the choice of tests and assessment of their assumptions, are provided in Supplementary Methods. A detailed exposition of data analysis methods for CRISPR screening, RNA sequencing (RNA-seq), ChIP-seq, and ATAC-seq is also provided in Supplementary Methods.

All other methods are described in detail in Supplementary Methods.

## Results

### Genome-wide screening identified ZFP91 as the leading activator of EVI1 expression

**Fig. 1:**
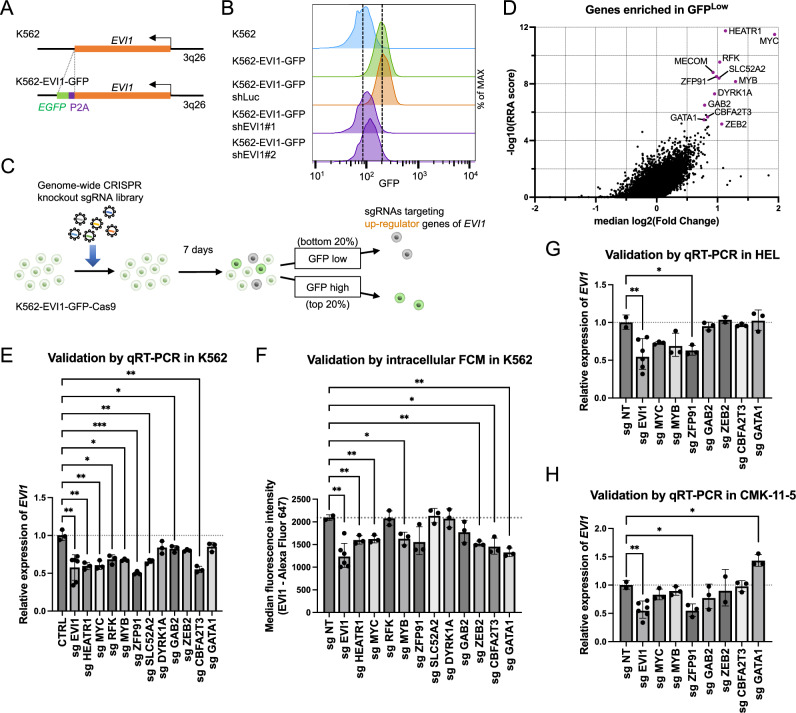
A CRISPR knockout screen identified ZFP91 as a leading candidate activator of EVI1 expression. **A** A schematic of GFP knock-in at the *EVI1* locus in K562 cells, with P2A self-cleavage sequence. **B** Flow cytometric analysis of GFP expression in K562-EVI1-GFP cells, compared to parental cells and those with *EVI1* knockdown via two different short hairpin RNAs. **C** Overview of the CRISPR/Cas9 screening process. A genome-wide knockout library was transduced into K562-EVI1-GFP-Cas9 cells at a multiplicity of infection of 0.3–0.4. After approximately 7 days of puromycin selection, the top and bottom 20% of transduced cells based on GFP intensity were sorted by fluorescent-activated cell sorting. **D** A scatter plot showing genes enriched in GFP^Low^ population from the CRISPR screen. The horizontal axis indicates median log2 fold change of sgRNAs for each gene relative to GFP^High^ fraction, while the vertical axis represents log2 modified robust ranking aggregation (RRA) score. Each dot corresponds to a gene, with purple dots indicating those with FDR < 1%. **E** *EVI1* mRNA expression levels measured by qRT-PCR in K562 cells after knockout of candidate genes. Expression levels were assessed 4 days post-transduction, with normalization to *PGK1* and *TBP*. **F** EVI1 protein levels assessed by intracellular flow cytometry using anti-EVI1 antibody in K562 cells after knockout of candidate genes. Median fluorescence intensity is shown as a measure of protein expression. **G**, **H** *EVI1* mRNA expression by qRT-PCR in HEL and CMK-11-5 cells. RNA was extracted 5 and 6 days post-transduction in HEL and CMK-11-5 cells, respectively. Expression levels were normalized to *GAPDH* and *YWHAZ* (HEL), or to *GAPDH* and *GUSB* (CMK-11-5). Each dot in (**E**–**H**) represents an individual sgRNA with different target sequences. All bar graphs depict geometric means ± geometric standard deviations. Statistical significance was determined by Welch’s analysis of variance (ANOVA), followed by unpaired Welch’s *t* test, on log-transformed values, with *p < 0.05, **p < 0.01, ***p < 0.001, ****p < 0.0001.

We first performed CRISPR/Cas9-mediated knock-in of porcine teschovirus-1 2A (P2A)-GFP sequence at the stop codon site of the *EVI1* gene as previously reported [14] in the human myeloid leukemia cell line K562 (Fig. 1A). K562 cells are a CML blast-phase cell line that is reported to express high levels of EVI1 [35] in the absence of 3q26 chromosomal aberrations detectable by the conventional G-band test [36]. We confirmed that GFP fluorescence intensity in our established K562-EVI1-GFP cell line was decreased by short hairpin RNA-mediated EVI1 knockdown (Fig. 1B).

Using this cell line, we performed GFP reporter-based CRISPR/Cas9 screening. Following transduction with the genome-scale CRISPR knock-out library, we collected the top and bottom 20% of transduced cells regarding GFP intensity by fluorescent-activated cell sorting (Fig. 1C). The transduced single gRNAs (sgRNAs) in both fractions were PCR-amplified, sequenced, and then computationally counted. We assumed genes enriched in the GFP^low^ fraction would be candidate activators of *EVI1* gene expression. Our screening listed 13, 17, and 48 top candidate genes with false discovery rate (FDR) < 1%, 5%, and 25%, respectively, as potential positive regulators of EVI1 expression. The top-ranked genes included *MECOM* and several ribosome-related genes, such as *HEAT repeat containing 1 (HEATR1)* and *pescadillo ribosomal biogenesis factor 1 (PES1)*, all of which were considered positive controls. We selected 11 genes as candidates and 2 genes (*MECOM* and *HEATR1*) as positive controls for validation (Fig. 1D and Supplementary Fig. 1A). Conversely, our screening also listed several candidates for negative regulators of EVI1 (Supplementary Fig. 1B, C), but we decided to focus on positive regulators in this study. We transduced the individual sgRNAs targeting these genes into Cas9-expressing K562-EVI1-GFP cells and assessed GFP fluorescence and EVI1 expression. All of the tested sgRNAs showed a tendency to decrease GFP intensity (Supplementary Fig. 1D) and *EVI1* mRNA expression (Fig. 1E), whereas only some of them reduced EVI1 protein levels (Fig. 1F).

We next verified whether these candidates regulate EVI1 expression in other EVI1-high myeloid leukemias in which 3q26 abnormalities have not been reported. We examined the human acute erythroid leukemia cell line HEL [37] and the human acute megakaryocytic leukemia cell line CMK-11-5 [38]. We evaluated *EVI1* mRNA levels by qRT-PCR in Cas9-expressing HEL and CMK-11-5 cells after sgRNA transduction. As a result, knockout of MYC, MYB, and ZFP91 commonly downregulated *EVI1* expression in HEL and CMK-11-5 cells, as well as K562 cells (Fig. 1G, H). We decided to focus on ZFP91 among them because ZFP91 knockout decreased EVI1 expression more significantly than the others in the three cell lines and because the loss of ZFP91 is less likely to affect normal hematopoietic stem cells than MYC or MYB, as there have been no detailed reports on ZFP91 in normal hematopoiesis.

### ZFP91 is the critical regulator of EVI1 expression

**Fig. 2:**
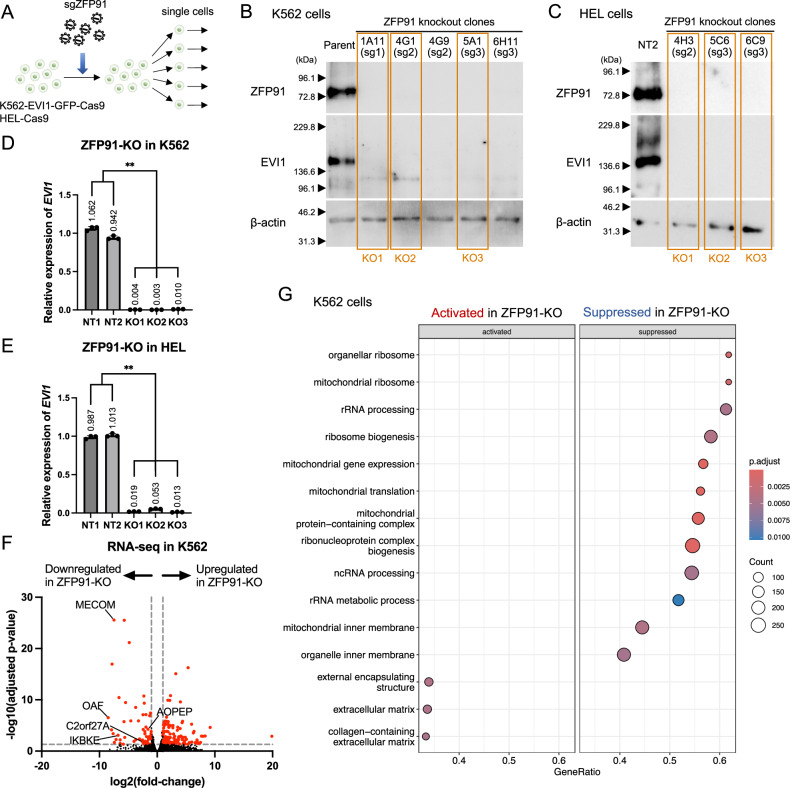
ZFP91 is the critical regulator of EVI1 expression. **A** Schematic representation of establishment of ZFP91-knockout clones from K562-EVI1-GFP and HEL cells. **B, C** EVI1 protein levels in ZFP91-knockout clones from K562-EVI1-GFP and HEL cells assessed by Western blotting. **D, E** *EVI1* mRNA expressions in ZFP91-knockout clones from K562-EVI1-GFP (**D**) and HEL cells (**E**) assessed by qRT-PCR. **F** Volcano plot showing RNA-seq data from ZFP91-knockout K562 cells and control K562 cells. The horizontal axis represents log2 fold change in gene expression. The vertical axis displays negative log10 of adjusted *p*-value. Each dot corresponds to a gene. Red dots indicate significantly upregulated or downregulated genes (|log2 FC| ≥ 1 and adjusted *p*-value < 0.05). Dashed lines denote significance thresholds. Notable genes are labeled for clarity. **G** GSEA results in GO terms from RNA-seq data comparing ZFP91-knockout K562 cells with control K562 cells, presented as a bubble plot. The vertical axis lists the enriched gene sets, while the horizontal axis represents gene ratio, indicating the proportion of differentially expressed genes within each gene set. The size of each bubble corresponds to the number of genes involved in that gene set. Bubble colors reflect adjusted *p*-values, with red indicating more significant gene sets (lower *p*-values) and blue representing less significant gene sets (higher *p*-values). All bar graphs depict geometric means ± geometric standard deviations. Statistical significance was determined by two-way repeated measures ANOVA with Geisser-Greenhouse correction on log-transformed relative expression, with *p < 0.05, **p < 0.01, ***p < 0.001, ****p < 0.0001.

Next, we established ZFP91 knockout clones in K562-EVI1-GFP cells and HEL cells by single-cell sorting after lentiviral sgRNA transduction (Fig. 2A). We evaluated EVI1 expression by qRT-PCR and Western blotting and found that EVI1 expression was reduced to almost undetectable levels in all ZFP91 knockout clones obtained (Fig. 2B–E). Conversely, ZFP91 overexpression induced a modest increase in EVI1 expression in K562 and HEL cells, whereas forced expression of ZFP91 did not substantially alter EVI1 levels in EVI1-high CMK-11-5 and F-36P cells or in EVI1-low THP-1 cells (Supplementary Fig. 1E, F), suggesting that ZFP91 is a necessary but not sufficient condition for EVI1 expression.

We then performed RNA-seq in the ZFP91-knockout K562-EVI1-GFP clones (KO1, KO2, KO3) and the control K562-EVI1-GFP-Cas9 cells transduced with non-targeting sgRNAs (NT1, NT2). Differential expression analysis revealed that *MECOM* was the most significantly downregulated gene by ZFP91 knockout among all annotated genes (Fig. 2F). Gene set enrichment analysis (GSEA) in Gene Ontology (GO) terms revealed that ribosome- or mitochondria-related genes were suppressed, and extracellular matrix-related genes were activated by ZFP91 knockout (Fig. 2G).

**Fig. 3:**
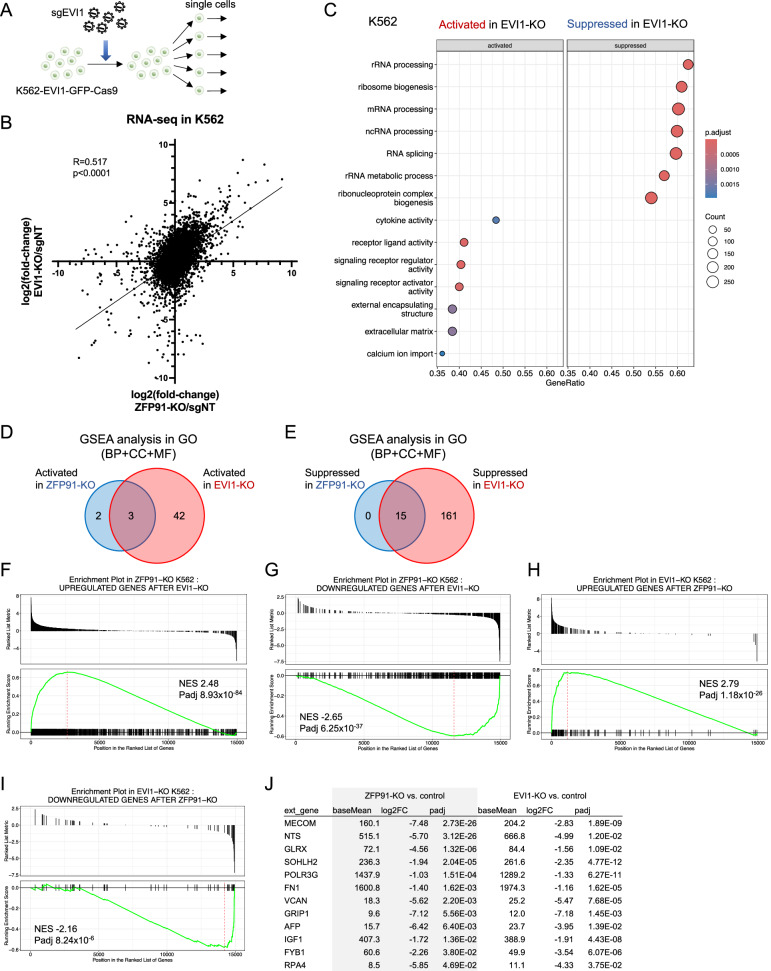
EVI1 knockout and ZFP91 knockout have similar downstream gene expression changes. **A** Schematic representation of the establishment of EVI1-knockout clones from K562-EVI1-GFP cells. **B** RNA-seq scatterplot analysis comparing the log2 fold-changes of mRNA levels after ZFP91 and EVI1 knockout in K562 cells. Each point corresponds to a gene. Correlation coefficient and *p*-value are indicated on the plot. **C** GSEA results in GO terms from RNA-seq data comparing EVI1-knockout K562 cells with control K562 cells, presented as a bubble plot. The plot format is the same as in Fig. 2G. **D**, **E** Venn diagrams showing enriched GO terms by GSEA in ZFP91-knockout and EVI1-knockout K562 cells with a cut-off *q* value of 0.05. See Supplementary Fig. 2A, B for specific names of common GO terms. **F–I** GSEA using custom gene sets consisting of differentially expressed genes (DEGs) by ZFP91 knockout or EVI1 knockout, with normalized enrichment scores (NES) and adjusted *p*-value indicated. The cut-off values of the DEGs were the same as in Fig. 2F. **J** Commonly downregulated genes by ZFP91 knockout and EVI1 knockout. “BaseMean” indicates relative read counts in control samples.

We hypothesized that the transcript changes by ZFP91 knockout and EVI1 knockout would be similar if the main target of ZFP91 is EVI1. To test this hypothesis, we generated EVI1-knockout K562-EVI1-GFP clones in the same way as ZFP91-knockout clones (Fig. 3A) and performed RNA-seq. As a result, fold changes in gene expression compared to controls were positively correlated between ZFP91 knockout and EVI1 knockout (*R* = 0.517) (Fig. 3B). Furthermore, GSEA in GO terms revealed that EVI1 knockout suppressed ribosome- or mitochondria-related genes and activated extracellular matrix-related genes like ZFP91 knockout (Fig. 3C). We checked all GO terms significantly changed in GSEA with a cutoff *q*-value of 0.05 and found that most of the activated or suppressed gene sets in ZFP91 knockout were similarly altered in EVI1 knockout (Fig. 3D, E and Supplementary Fig. 2A, B).

We also performed GSEA using custom gene sets consisting of genes differentially expressed by ZFP91 knockout or EVI1 knockout. As a result, 547 downregulated genes or 1030 upregulated genes by EVI1 knockout with a cutoff value of adjusted *p*-value < 0.05 and absolute log2 fold change ≥ 1 were identically downregulated or upregulated by ZFP91 knockout, respectively (Fig. 3F, G). Similarly, 57 downregulated genes or 119 upregulated genes by ZFP91 knockout with the same cutoff value were identically downregulated or upregulated by EVI1 knockout, respectively (Fig. 3H, I). These results suggest that ZFP91 is essential for EVI1 expression at the transcriptional level and that EVI1 is one of the primary target genes of ZFP91.

Notably, ZFP91 knockout resulted in far fewer differentially expressed genes (DEGs) than EVI1 knockout, despite the general expectation that disruption of upstream genes has a greater impact than disruption of downstream genes. One possible explanation is that ZFP91 knockout markedly reduces EVI1 expression but leaves a small amount of residual EVI1 expression, which may attenuate the genome-wide transcriptional changes observed upon EVI1 knockout. We extracted commonly downregulated genes by ZFP91 knockout and EVI1 knockout (Fig. 3J). Among them, *insulin-like growth factor 1 (IGF1), fibronectin 1 (FN1), and versican (VCAN)* are reported to be related to epithelial-to-mesenchymal transition (EMT) programs [39–41]. These genes may represent key downstream effectors in the ZFP91–EVI1 axis, in consideration of previous reports linking EVI1 to refractoriness via EMT-like programs [42].

### ZFP91 binds to the EVI1 promoter and activates the promoter

ZFP91 contains five C2H2-type zinc finger (ZnF) domains that mediate its interactions with diverse substrate proteins and regulate their ubiquitination. Notably, the ZnF domain required for substrate recognition varies by target: ZnF2 for NIK [25], ZnF3 for FOXA1 [43], and ZnF4 for CRBN [26], as previously reported. To determine whether the EVI1 reduction in ZFP91-deficient cells results from loss of ubiquitination of a specific substrate, we tested whether ZFP91 mutants lacking individual ZnF domains could rescue EVI1 expression. Using an sgZFP91#3-resistant wild-type ZFP91 construct as a template, we generated five individual cysteine-to-alanine ZnF mutants and introduced them into the HEL ZFP91-KO3 clone. Unlike wild-type ZFP91, none of the mutants fully restored EVI1 mRNA or protein (Supplementary Fig. 2C, E). These findings indicate that all ZnF domains are required to maintain EVI1 expression, arguing against a model in which a single ZnF-dependent interaction explains the ZFP91–EVI1 regulatory axis.

**Fig. 4:**
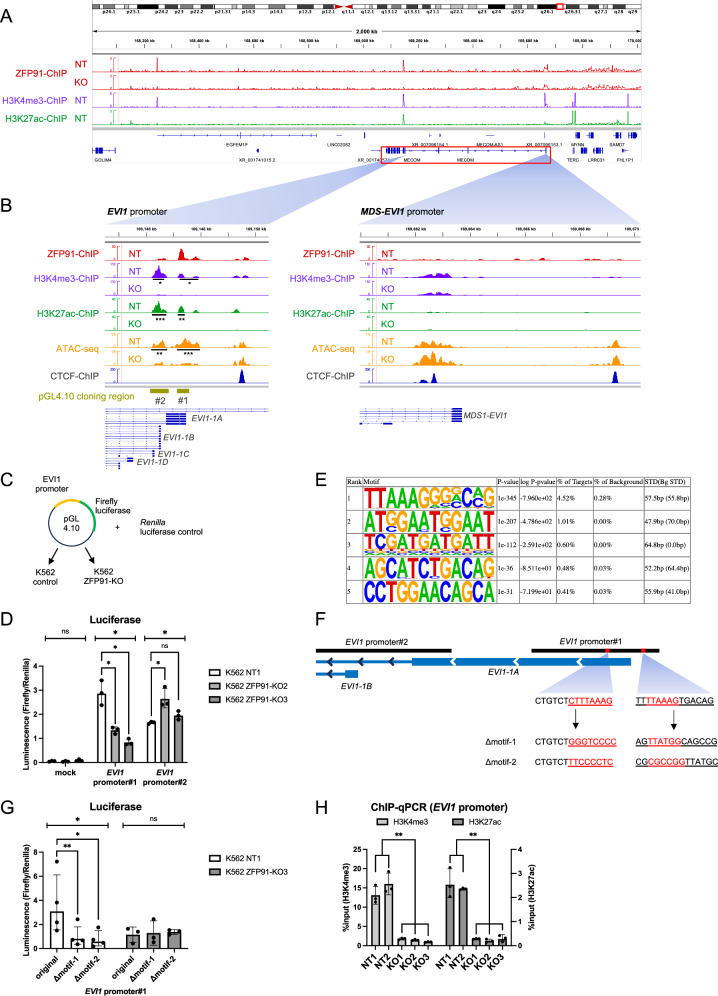
ZFP91 binds to the *EVI1* promoter and positively regulates the promoter activity. **A** Genome browser view around the *MECOM* gene region (±2 kb) showing ChIP-seq for ZFP91, H3K4me3, and H3K27ac in control K562 (NT) cells. The vertical axis represents fold enrichment of read counts relative to input per bin. To confirm ZFP91 peak specificity, ChIP-seq for ZFP91 in ZFP91-knockout K562 (KO) cells is included. **B** Expanded view of the *EVI1* and *MDS1-EVI1* promoter regions from (**A**), showing ZFP91 ChIP-seq in NT cells, along with ChIP-seq for H3K4me3 and H3K27ac, and ATAC-seq in both NT and KO cells, and with ChIP-seq for CTCF in K562 cells from public sources. The vertical axis for ATAC-seq shows pile-up read counts per million per bin. The genomic locations used in subsequent luciferase promoter assays are indicated. Asterisks (*) denote statistically significant differences in peak height, assessed using MAnorm2. **C** Schematic illustrating luciferase reporter assay designed to measure promoter activity. **D** Results of the luciferase reporter assays assessing the activity of *EVI1* promoter#1 and promoter#2 constructs transfected into NT and KO cells. Cells were collected 72 h post-transfection. *Renilla* luciferase activity was used to normalize transfection efficiency. **E** Motif analysis using ChIP-seq for ZFP91 in NT cells. **F** A schematic of ZFP91 motif destruction in *EVI1* promoter#1 for subsequent assay. The red characters indicate the main motif TTAAAG, and the underlines indicate replaced bases. **G** Results of luciferase reporter assays to measure the activity of *EVI1* promoter#1 with disrupted ZFP91 motifs in NT and KO cells. **H** Results of ChIP-qPCR for H3K4me3 and H3K27ac targeting the *EVI1* promoter in NT and KO cells. All bar graphs depict geometric means ± geometric standard deviations. Statistical significance in the luciferase assay was assessed using log-transformed Firefly luminescence values normalized to *Renilla*. One-way repeated measures ANOVA with Geisser-Greenhouse correction was first performed separately for each group, with *p*-values adjusted for multiple testing using the Holm-Bonferroni method. Dunnett’s test was then applied for post hoc comparisons in groups showing significant ANOVA result. Statistical significance in ChIP-qPCR was assessed using log-transformed %input values. Two-way repeated measures ANOVA with Geisser-Greenhouse correction was performed separately for each group (H3K4me3 and H3K27ac), with *p*-values adjusted for multiple testing using the Holm-Bonferroni method. *p < 0.05, **p < 0.01, ***p < 0.001, ****p < 0.0001.

Since ZFP91 is reported to function as a transcription factor [26], we next performed ChIP-seq for ZFP91, histone H3 lysine 4 trimethylation (H3K4me3), and histone H3 lysine 27 acetylation (H3K27ac) in ZFP91-knockout K562 clones (KO1, KO2, KO3) and control cells (NT1, NT2). As a result, we listed a total of 1917 peaks across the genome by ChIP-seq for ZFP91 in control cells. We detected distinct ZFP91 peaks in the *EVI1* promoter, suggesting that ZFP91 binds to the *EVI1* promoter. No apparent ZFP91 peaks were detected within the previously reported topologically associated domain (TAD) containing *EVI1* [44, 45], including the *MDS1* promoter region. The nearest ZFP91 peak was located 900 kb away from the *EVI1* promoter (Fig. 4A), outside this TAD.

Human *EVI1* has several mRNA splicing variants with different transcription start sites (TSSs), and these variants are mainly categorized into *EVI1-1A, -1B, -1C, -1D*, and *-3L* according to the TSS position [22]. Two major peak regions were detected near the *EVI1* TSSs by our ChIP-seq for H3K4me3 and H3K27ac in control K562 cells, consistent with the previous reports [46]. We named one peak near the *EVI1-1A* TSS as *EVI1* promoter#1 and the other near the TSSs of *EVI1-1B* and *-1C* as *EVI1* promoter#2. *EVI1* promoter#2 contains the previously reported 318 bp minimal promoter of *EVI1* [22]. Interestingly, the central ZFP91 peak was located in *EVI1* promoter#1, whereas no significant ZFP91 peaks were detected in *EVI1* promoter#2 (Fig. 4B).

We cloned the *EVI1* promoter#1 and *EVI1* promoter#2 sequences into the pGL4.10 luciferase expression vector and performed luciferase promoter assay in ZFP91-knockout K562 (KO2 and KO3) and control cells (NT1) (Fig. 4C). As expected, *EVI1* promoter#1 activity was significantly reduced by ZFP91 knockout, whereas *EVI1* promoter#2 activity was not reduced (or slightly increased) by ZFP91 knockout (Fig. 4D).

We further performed motif analysis on our ChIP-seq data for ZFP91 (Fig. 4E). The most characteristic ZFP91 motif sequence, TTAAAG, appeared three times in two places within *EVI1* promoter#1. We replaced the ZFP91 motif sequences in the *EVI1* promoter#1 on the pGL4.10 vector with two different random sequences (Fig. 4F) and performed luciferase promoter assay again. As a result, modification of ZFP91 motifs in *EVI1* promoter#1 significantly reduced promoter activity in control K562 cells, whereas ZFP91-knockout K562 cells showed no significant reduction in promoter activity by ZFP91 motif modification, as we expected (Fig. 4G). These results suggest that ZFP91 binds to the *EVI1* promoter near the *EVI1-1A* TSS and directly regulates the promoter activity.

Our ChIP-seq analysis also showed that active histone modifications (H3K4me3 and H3K27ac) were significantly reduced by ZFP91 knockout in the *EVI1* promoter but not in the *MDS1* promoter (Fig. 4B). ChIP-quantitative PCR (ChIP-qPCR) estimated that ZFP91 knockout reduced H3K4me3 and H3K27ac in the *EVI1* promoter by approximately 90% (Fig. 4H). Interestingly, the reduction of active histone marks was observed in both *EVI1* promoter#1 and *EVI1* promoter#2, although direct binding of ZFP91 was only suggested in *EVI1* promoter#1 (Fig. 4B). We also performed ATAC-seq in ZFP91-knockout and control K562 cells. Similarly to ChIP-seq for H3K4me3 and H3K27ac, ZFP91 knockout significantly reduced accessibility to the *EVI1* promoter in both *EVI1* promoter#1 and *EVI1* promoter#2 (Fig. 4B).

### EVI1 is one of the major targets of ZFP91

**Fig. 5:**
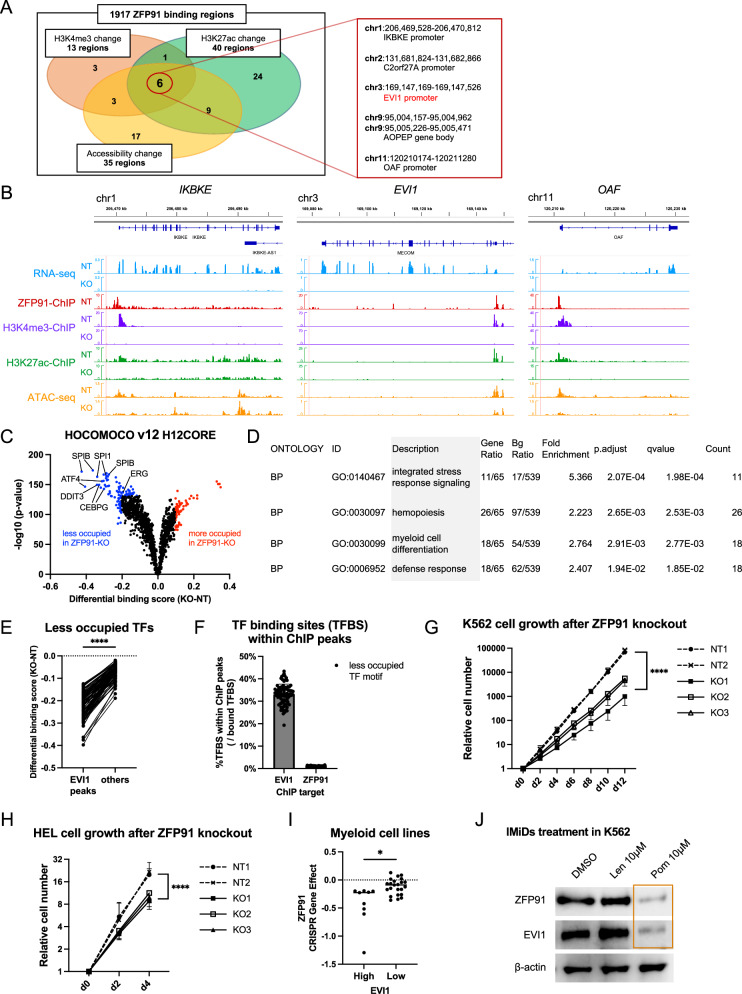
ZFP91 alters genome-wide transcriptional network through EVI1. **A** Venn diagram showing the number of ZFP91 peak regions with significant epigenetic changes by ZFP91 knockout. Six ZFP91 peaks showed significant changes in both H3K4me3, H3K27ac, and chromatin accessibility (ATAC) by MAnorm2 with *p*-value < 0.05. **B** Genome browser view of the regions around the predicted target genes of ZFP91 indicated in (**A**) with stacked mapped reads for RNA-seq and ATAC-seq, and fold enrichment for ChIP-seq. **C** Results of the footprinting analysis on ATAC-seq using HOCOMOCO v12 CORE database. The plot shows differential binding score for each transcription factor (TF) motif on the horizontal axis, and negative log10 of *p*-value on the vertical axis. Each dot corresponds to a motif. **D** GO enrichment analysis for TFs with less-occupied motifs by ZFP91 knockout compared to all TFs in HOCOMOCO v12 CORE database. TFs without ENTREZ ID were excluded from the analysis. **E** Comparison of differential binding scores between the EVI1-ChIP peak regions and other regions, regarding the less-occupied TF motifs in ZFP91-KO, as indicated by the blue plots in (**C**). **F** Proportion of TF binding sites (TFBS) in EVI1-ChIP peak region or ZFP91-ChIP peak region, regarding the less-occupied TF motifs in ZFP91-KO. The bar graph depicts means ± standard deviations. **G, H** Cell growth after ZFP91 knockout in K562 (**G**) and HEL (**H**) cells. The bar graph depicts geometric means ± geometric standard deviations. **I** Dependency of myeloid leukemia cell lines on ZFP91 from the public database DepMap. The vertical axis represents the dependency score; the smaller the score, the higher the dependence. Dots represent individual cell lines, and horizontal bars indicate the mean. The cutoff values for EVI1-low and EVI1-high expression levels were set to 1.0 and 3.0, respectively. The expression level in DepMap is defined as log2 of “total read counts per million (TPM) + 1”. **J** ZFP91 and EVI1 protein expression in K562 cells treated with DMSO, lenalidomide, or pomalidomide for 4 days. Statistical significance was determined by paired *t*-test for (**E**) and Welch’s *t-*test for (**I**). Significant differences in cell proliferation rates between groups (NT vs. KO) were assessed for (**G**, **H**) using a linear mixed-effects model with log-transformed relative cell counts, including day and the interaction between day and group as fixed effects, and experimental batch as a random intercept. *p < 0.05, **p < 0.01, ***p < 0.001, ****p < 0.0001.

We assessed how prevalent the regions are, where all three signals (H3K4me3, H3K27ac, and accessibility) decrease within ZFP91 peaks, similar to the *EVI1* promoter, on a genome-wide basis. Quantitative comparison of ZFP91-knockout and control K562 cells with peak signals from ChIP-seq for H3K4me3 and H3K27ac and from ATAC-seq revealed that only six regions related to five genes *(Inhibitor of nuclear factor kappa B kinase subunit epsilon (IKBKE), Chromosome 2 open reading frame 27A (C2orf27A), MECOM(EVI1), Aminopeptidase O (putative) (AOPEP), Out at first homolog (OAF))* showed statistically significant changes in all three signals within ZFP91 peaks (Fig. 5A). RNA-seq revealed that RNA expression levels of all five genes, especially the four genes with ZFP91 peak in the promoter, were significantly reduced by ZFP91 knockout but not by EVI1 knockout (Figs. 2F, 5B). Notably, *IKBKE* was extracted as a direct downstream target of ZFP91 since it has already been reported that ZFP91 activates the nuclear factor-kappa B pathway, binding to the *IKBKE* promoter and upregulating its expression [26]. These data suggest that the transcriptional target genes of ZFP91 were minimal and that *EVI1* is one of the primary target genes of ZFP91.

We hypothesized that ZFP91 affects the genome-wide transcriptional network related to EVI1 because the direct target genes of ZFP91 were minimal. To test this hypothesis, we performed a footprinting analysis on ATAC-seq data with two different motif databases. We detected transcription factors that were less or more occupied in ZFP91-knockout K562 cells compared to controls (Fig. 5C and Supplementary Fig. 3A). GO enrichment analysis revealed that transcription factors whose binding scores were significantly reduced by ZFP91 knockout were enriched for genes involved in stress response, hematopoiesis, and myeloid differentiation (Fig. 5D). These included known targets of EVI1, such as ETS transcription factor ERG (ERG) [12] and Spi-1 proto-oncogene (SPI1) [47], which is consistent with our hypothesis (Supplementary Fig. 3B).

Therefore, we asked whether or not the change in the binding status of these transcription factors occurred in the genomic regions targeted by EVI1. EVI1-ChIP peaks were determined as the 17,879 regions identified by analyzing public ChIP-seq data for EVI1 in K562 cells. We compared the distribution of binding score differences (KO - NT) between inside and outside EVI1-ChIP peaks for the less occupied motifs in ZFP91-knockout K562 cells. As a result, the binding score differences were more significant inside EVI1-ChIP peaks than outside (Fig. 5E). Most ZFP91-ChIP peaks did not overlap with motif regions bound by transcription factors, whereas about one-third of EVI1-ChIP peaks overlapped with them (Fig. 5F). These results support our hypothesis that ZFP91 alters the transcriptional network through EVI1 rather than directly.

### Therapeutic potential of ZFP91 in EVI1-associated leukemia

Consistent with previous reports, knockout of ZFP91 in K562 and HEL cells significantly reduced cell proliferation (Fig. 5G, H). However, EVI1 knockout did not affect cell growth in K562 cells (Supplementary Fig. 3C). The public database showed most of the EVI1-high myeloid leukemia cell lines showed little dependency on EVI1 [48], while dependency on ZFP91 is significantly higher in EVI1-high myeloid leukemia cell lines than in EVI1-low myeloid leukemia cell lines (Fig. 5I). These data suggest that in these EVI1-associated leukemias, targeting ZFP91 may be more effective than targeting EVI1 itself. We suggest that ZFP91 may play a role in the entire transcriptional system that overexpresses EVI1.

## Discussion

To our knowledge, this is the first report of a genome-wide screening for regulatory factors of EVI1. Through unbiased screening, we have identified that ZFP91 is a potent EVI1 regulator in at least selected cases of EVI1-associated leukemias. We found that knockout of ZFP91 in such leukemias resulted in an almost complete loss of EVI1 expression at both mRNA and protein levels. We also demonstrated in K562 cells that ZFP91 binds to the *EVI1* promoter and works as an essential factor for maintaining active histone marks and chromatin accessibility at the *EVI1* promoter. ChIP-seq and ATAC-seq analysis also suggested that transcriptional targets of ZFP91 were relatively limited and that EVI1 plays a central role in controlling the transcriptional network downstream of ZFP91.

Our screening approach for upstream regulators was remarkably simple: we knocked in a fluorescent reporter at the endogenous locus of the target gene *EVI1* and performed a genome-wide CRISPR knockout. Although previous studies have employed reporter-based screening to identify transcriptional regulators, these approaches typically introduce only the promoter sequence of the target gene along with a fluorescent reporter [49, 50], rather than knocking the reporter into the endogenous locus. Such strategies are less suited to capturing regulators that act through the broader epigenomic context. Other studies have used custom libraries limited to transcription factors [51, 52] rather than the whole genome. Performing a genome-wide screen offers several advantages, including the ability to identify positive controls such as ribosomal genes and to uncover transcriptional regulators beyond classical transcription factors.

As previously reported, ZFP91 knockdown induces apoptosis in AML [27], suggesting that ZFP91 is a potential therapeutic target for myeloid leukemia. Taken together with our results, targeting ZFP91 may improve treatment response in some EVI1-associated leukemias by unleashing the EVI1-high-expression status, although further in vivo validation is needed. Targeting ZFP91 is relatively feasible because napabucasin-based proteolysis targeting chimera (PROTAC) or immunoregulatory drugs (IMiDs) have been reported to degrade ZFP91 [53, 54]. Indeed, K562 cells treated with pomalidomide showed significantly reduced levels of ZFP91 along with EVI1 (Fig. 5J).

Beyond hematologic malignancies, ZFP91 expression has been linked to prognosis in several solid tumors in a tumor-type-dependent manner [29, 55]. Likewise, EVI1 (MECOM) overexpression has been reported in multiple solid tumors, including colorectal, pancreatic, and hepatocellular carcinomas, where it is associated with aggressive disease and poor prognosis [56–58]. In hepatocellular carcinoma, EVI1 upregulation has been primarily attributed to genomic amplification of the 3q26 locus [58]. However, public ChIP-seq data from the hepatocellular carcinoma cell line HepG2 reveal ZFP91 binding at the *EVI1* promoter (Supplementary Fig. 3F), similar to that observed in K562 cells, suggesting a ZFP91–EVI1 regulatory axis may operate independently of 3q26 copy-number gain. Thus, the ZFP91–EVI1 axis identified in myeloid leukemia may represent a broader transcriptional mechanism relevant to diverse malignancies.

A recent study demonstrated that the *EVI1* promoter-proximal site that is involved in CTCF-mediated looping is important for *EVI1* expression via long-range chromatin interaction in myeloid leukemias without 3q rearrangement [46]. Consistent with this, our ZFP91-knockout K562 cells showed relatively decreased levels of H3K4me3 and H3K27ac in the *EVI1* promoter-proximal CTCF-binding site compared to controls (Fig. 3B), indicating that ZFP91 might influence *EVI1* enhancer activity. On the other hand, while it has been reported that *EVI1* distal enhancer exists around the *MDS1* promoter region [46], neither H3K4me3, H3K27ac, nor ATAC signals showed significant changes around the *MDS1* promoter by ZFP91 knockout. Further studies are needed to elucidate the overall mechanism of EVI1 expression by ZFP91.

We must admit that cell-line-based experiments on EVI1-associated leukemia have a lot of limitations. It has been pointed out that most AML cell lines with high EVI1 expression do not depend on EVI1 expression for their survival [48], despite the dismal outcome of EVI1-associated AML in clinical settings. The reason for such a crucial difference between clinical and in vitro settings has not been elucidated so far, and this is one of the major obstacles to research on EVI1-associated AML. Consistent with this, our EVI1 knockout K562 also showed no significant changes in proliferation (Supplementary Fig. 3C). Such independency on EVI1 in vitro paradoxically made it possible our reporter-based knockout screening because lowering GFP intensity can be detected only from alive cells. We also acknowledge that our detailed analyses were mainly performed in K562 cells, which is a limitation of this study. However, ZFP91-dependent regulation of EVI1 was also observed in another EVI1-expressing AML cell line, HEL, suggesting that this mechanism is not restricted to K562 cells. Our findings need to be confirmed in the future using a mouse model that mimics EVI1-associated myeloid leukemia, but there are a limited number of stably EVI1-high AML mouse models that do not involve induction of a 3q26 chromosomal translocation or forced expression of EVI1 itself, which requires the development of experimental systems.

While ZFP91 expression was reported to be higher in AML than in normal peripheral blood mononuclear cells [24], public databases show no apparent correlation between EVI1 and ZFP91 expression in AML patients (Supplementary Fig. 3D, E). This suggests that ZFP91 is not a single factor that determines EVI1 activation, but plays an essential role in maintaining EVI1 activation at least in a subset of EVI1-related leukemias. However, in terms of the search for therapeutic targets, the significant fact is that we have identified a factor with low impact on normal hematopoiesis that has the potential to reverse EVI1 overexpression in a specific patient population. The effect of ZFP91 on normal hematopoiesis is thought to be limited, since ZFP91-knockout mice are reported to be non-fetal lethal [28], while the fetuses of EVI1-knockout mice exhibit a marked decrease in hematopoietic stem cells and die during fetal life [3]. These findings raise a possibility that EVI1 regulation by ZFP91 is leukemia-specific. This hypothesis should be tested in the future by analyzing leukemia generated by the KMT2A fusion protein, which has already been reported to bind to the *EVI1* promoter [18]. Further investigation is needed to determine if ZFP91 is a potential therapeutic target for EVI1-related myeloid leukemia.

## Supplementary information

Supplemental Document

Supplementary Figure 1

Supplementary Figure 2

Supplementary Figure 3

Supplementary Table 1

Supplementary Table 2

## Data Availability

The RNA-seq, ChIP-seq, and ATAC-seq generated in this study are available in NCBI Sequence Read Archive (SRA) (RRID:SCR_004891) at PRJDB19903, and the corresponding processed data are available in Genomic Expression Archive (GEA) at E-GEAD-891, E-GEAD-900, and E-GEAD-893. Sources of public database are described in Supplementary Methods.

## References

[1] Kim DDH, Novitzky Basso I, Kim TS, Yi SY, Kim KH, Murphy T, et al. The 17-gene stemness score associates with relapse risk and long-term outcomes following allogeneic haematopoietic cell transplantation in acute myeloid leukaemia. EJHaem. 2022;3:873–84. 36051057 10.1002/jha2.466 PMC9422016

[2] Thoms JAI, Beck D, Pimanda JE. Transcriptional networks in acute myeloid leukemia. Genes Chromosomes Cancer. 2019;58:859–74. 31369171 10.1002/gcc.22794

[3] Goyama S, Yamamoto G, Shimabe M, Sato T, Ichikawa M, Ogawa S, et al. Evi-1 is a critical regulator for hematopoietic stem cells and transformed leukemic cells. Cell Stem Cell. 2008;3:207–20. 18682242 10.1016/j.stem.2008.06.002

[4] Kataoka K, Sato T, Yoshimi A, Goyama S, Tsuruta T, Kobayashi H, et al. Evi1 is essential for hematopoietic stem cell self-renewal, and its expression marks hematopoietic cells with long-term multilineage repopulating activity. J Exp Med. 2011;208:2403–16. 22084405 10.1084/jem.20110447 PMC3256960

[5] Wu X, Wang H, Deng J, Zheng X, Ling Y, Gong Y. Prognostic significance of the EVI1 gene expression in patients with acute myeloid leukemia: a meta-analysis. Ann Hematol. 2019;98:2485–96. 31482295 10.1007/s00277-019-03774-z

[6] Laricchia-Robbio L, Nucifora G. Significant increase of self-renewal in hematopoietic cells after forced expression of EVI1. Blood Cells Mol Dis. 2008;40:141–7. 17913523 10.1016/j.bcmd.2007.07.012 PMC2323624

[7] Kustikova OS, Schwarzer A, Stahlhut M, Brugman MH, Neumann T, Yang M, et al. Activation of Evi1 inhibits cell cycle progression and differentiation of hematopoietic progenitor cells. Leukemia. 2013;27:1127–38. 23212151 10.1038/leu.2012.355

[8] Buonamici S, Li D, Chi Y, Zhao R, Wang X, Brace L, et al. EVI1 induces myelodysplastic syndrome in mice. J Clin Investig. 2004;114:713–9. 15343390 10.1172/JCI21716 PMC514587

[9] Yoshimi A, Goyama S, Watanabe-Okochi N, Yoshiki Y, Nannya Y, Nitta E, et al. Evi1 represses PTEN expression and activates PI3K/AKT/mTOR via interactions with polycomb proteins. Blood. 2011;117:3617–28. 21289308 10.1182/blood-2009-12-261602

[10] Kurokawa M, Mitani K, Irie K, Matsuyama T, Takahashi T, Chiba S, et al. The oncoprotein Evi-1 represses TGF-beta signalling by inhibiting Smad3. Nature. 1998;394:92–6. 9665135 10.1038/27945

[11] Xu X, Woo CH, Steere RR, Lee BC, Huang Y, Wu J, et al. EVI1 acts as an inducible negative-feedback regulator of NF-kappaB by inhibiting p65 acetylation. J Immunol. 2012;188:6371–80. 22581859 10.4049/jimmunol.1103527 PMC3370108

[12] Masamoto Y, Chiba A, Mizuno H, Hino T, Hayashida H, Sato T, et al. EVI1 exerts distinct roles in AML via ERG and cyclin D1 promoting a chemoresistant and immune-suppressive environment. Blood Adv. 2023;7:1577–93. 36269819 10.1182/bloodadvances.2022008018 PMC10139867

[13] Yamazaki H, Suzuki M, Otsuki A, Shimizu R, Bresnick EH, Engel JD, et al. A remote GATA2 hematopoietic enhancer drives leukemogenesis in inv(3)(q21;q26) by activating EVI1 expression. Cancer Cell. 2014;25:415–27. 24703906 10.1016/j.ccr.2014.02.008 PMC4012341

[14] Smeenk L, Ottema S, Mulet-Lazaro R, Ebert A, Havermans M, Varea AA, et al. Selective requirement of MYB for oncogenic hyperactivation of a translocated enhancer in leukemia. Cancer Discov. 2021;11:2868–83. 33980539 10.1158/2159-8290.CD-20-1793 PMC8563373

[15] Ottema S, Mulet-Lazaro R, Erpelinck-Verschueren C, van Herk S, Havermans M, Arricibita Varea A, et al. The leukemic oncogene EVI1 hijacks a MYC super-enhancer by CTCF-facilitated loops. Nat Commun. 2021;12:5679. 34584081 10.1038/s41467-021-25862-3 PMC8479123

[16] Nucifora G, Birn DJ, Espinosa R, Erickson P, LeBeau MM, Roulston D, et al. Involvement of the AML1 gene in the t(3;21) in therapy-related leukemia and in chronic myeloid leukemia in blast crisis. Blood. 1993;81:2728–34. 8490181

[17] Raynaud SD, Baens M, Grosgeorge J, Rodgers K, Reid CD, Dainton M, et al. Fluorescence in situ hybridization analysis of t(3; 12)(q26; p13): a recurring chromosomal abnormality involving the TEL gene (ETV6) in myelodysplastic syndromes. Blood. 1996;88:682–9. 8695816

[18] Arai S, Yoshimi A, Shimabe M, Ichikawa M, Nakagawa M, Imai Y, et al. Evi-1 is a transcriptional target of mixed-lineage leukemia oncoproteins in hematopoietic stem cells. Blood. 2011;117:6304–14. 21190993 10.1182/blood-2009-07-234310

[19] Gröschel S, Lugthart S, Schlenk RF, Valk PJ, Eiwen K, Goudswaard C, et al. High EVI1 expression predicts outcome in younger adult patients with acute myeloid leukemia and is associated with distinct cytogenetic abnormalities. J Clin Oncol. 2010;28:2101–7. 20308656 10.1200/JCO.2009.26.0646

[20] Tang Z, Wang W, Yang S, El Achi H, Fang H, Nahmod KA. et al. 3q26.2/MECOM rearrangements by pericentric Inv(3): diagnostic challenges and clinicopathologic features. Cancers. 2023;15:458 36672407 10.3390/cancers15020458 PMC9856433

[21] Qin YZ, Zhao T, Zhu HH, Wang J, Jia JS, Lai YY, et al. High EVI1 expression predicts poor outcomes in adult acute myeloid leukemia patients with intermediate cytogenetic risk receiving chemotherapy. Med Sci Monit. 2018;24:758–67. 29408852 10.12659/MSM.905903 PMC5810369

[22] Maicas M, Vazquez I, Vicente C, Garcia-Sanchez MA, Marcotegui N, Urquiza L, et al. Functional characterization of the promoter region of the human EVI1 gene in acute myeloid leukemia: RUNX1 and ELK1 directly regulate its transcription. Oncogene. 2013;32:2069–78. 22689058 10.1038/onc.2012.222

[23] Manachai N, Saito Y, Nakahata S, Bahirvani AG, Osato M, Morishita K. Activation of EVI1 transcription by the LEF1/β-catenin complex with p53-alteration in myeloid blast crisis of chronic myeloid leukemia. Biochem Biophys Res Commun. 2017;482:994–1000. 27908728 10.1016/j.bbrc.2016.11.146

[24] Unoki M, Okutsu J, Nakamura Y. Identification of a novel human gene, ZFP91, involved in acute myelogenous leukemia. Int J Oncol. 2003;22:1217–23. 12738986

[25] Jin X, Jin HR, Jung HS, Lee SJ, Lee JH, Lee JJ. An atypical E3 ligase zinc finger protein 91 stabilizes and activates NF-kappaB-inducing kinase via Lys63-linked ubiquitination. J Biol Chem. 2010;285:30539–47. 20682767 10.1074/jbc.M110.129551 PMC2945548

[26] Wu W, Nelson GM, Koch R, Donovan KA, Nowak RP, Heavican-Foral TB, et al. Overcoming IMiD resistance in T-cell lymphomas through potent degradation of ZFP91 and IKZF1. Blood. 2022;139:2024–37. 34936696 10.1182/blood.2021014701 PMC8972091

[27] Zhang Z, Zhong L, Dan W, Chu X, Liu C, Luo X, et al. ZFP91 promotes cell proliferation and inhibits cell apoptosis in AML via inhibiting the proteasome-dependent degradation of RIP1. Int J Med Sci. 2022;19:274–85. 35165513 10.7150/ijms.67436 PMC8795797

[28] Mi C, Wang Z, Li MY, Zhang ZH, Ma J, Jin X. Zinc finger protein 91 positively regulates the production of IL-1β in macrophages by activation of MAPKs and non-canonical caspase-8 inflammasome. Br J Pharmacol. 2018;175:4338–52. 30182366 10.1111/bph.14493 PMC6240129

[29] Ma J, Mi C, Wang KS, Lee JJ, Jin X. Zinc finger protein 91 (ZFP91) activates HIF-1α via NF-κB/p65 to promote proliferation and tumorigenesis of colon cancer. Oncotarget. 2016;7:36551–62. 27144516 10.18632/oncotarget.9070 PMC5095020

[30] Sanjana NE, Shalem O, Zhang F. Improved vectors and genome-wide libraries for CRISPR screening. Nat Methods. 2014;11:783–4. 25075903 10.1038/nmeth.3047 PMC4486245

[31] Joung J, Konermann S, Gootenberg JS, Abudayyeh OO, Platt RJ, Brigham MD, et al. Genome-scale CRISPR-Cas9 knockout and transcriptional activation screening. Nat Protoc. 2017;12:828–63. 28333914 10.1038/nprot.2017.016 PMC5526071

[32] Vandesompele J, De Preter K, Pattyn F, Poppe B, Van Roy N, De Paepe A, et al. Accurate normalization of real-time quantitative RT-PCR data by geometric averaging of multiple internal control genes. Genome Biol. 2002;3:RESEARCH0034. 10.1186/gb-2002-3-7-research0034 PMC126239 12184808

[33] Pfaffl MW, Tichopad A, Prgomet C, Neuvians TP. Determination of stable housekeeping genes, differentially regulated target genes and sample integrity: bestKeeper-excel-based tool using pair-wise correlations. Biotechnol Lett. 2004;26:509–15. 15127793 10.1023/b:bile.0000019559.84305.47

[34] Grandi FC, Modi H, Kampman L, Corces MR. Chromatin accessibility profiling by ATAC-seq. Nat Protoc. 2022;17:1518–52. 35478247 10.1038/s41596-022-00692-9 PMC9189070

[35] Fichelson S, Dreyfus F, Berger R, Melle J, Bastard C, Miclea JM, et al. Evi-1 expression in leukemic patients with rearrangements of the 3q25-q28 chromosomal region. Leukemia. 1992;6:93–99. 1552747

[36] Naumann S, Reutzel D, Speicher M, Decker HJ. Complete karyotype characterization of the K562 cell line by combined application of G-banding, multiplex-fluorescence in situ hybridization, fluorescence in situ hybridization, and comparative genomic hybridization. Leuk Res. 2001;25:313–22. 11248328 10.1016/s0145-2126(00)00125-9

[37] Mackinnon RN, Wall M, Zordan A, Nutalapati S, Mercer B, Peverall J, et al. Genome organization and the role of centromeres in evolution of the erythroleukaemia cell line HEL. Evol Med Public Health. 2013;2013:225–40. 24481202 10.1093/emph/eot020 PMC3868402

[38] Iwamoto K, Bundo M, Ueda J, Nakano Y, Ukai W, Hashimoto E, et al. Detection of chromosomal structural alterations in single cells by SNP arrays: a systematic survey of amplification bias and optimized workflow. PLoS ONE. 2007;2:e1306. 18074030 10.1371/journal.pone.0001306 PMC2111048

[39] Zhao C, Wang Q, Wang B, Sun Q, He Z, Hong J, et al. IGF-1 induces the epithelial-mesenchymal transition via Stat5 in hepatocellular carcinoma. Oncotarget. 2017;8:111922–30. 29340101 10.18632/oncotarget.22952 PMC5762369

[40] Li B, Shen W, Peng H, Li Y, Chen F, Zheng L, et al. Fibronectin 1 promotes melanoma proliferation and metastasis by inhibiting apoptosis and regulating EMT. Onco Targets Ther. 2019;12:3207–21. 31118673 10.2147/OTT.S195703 PMC6503329

[41] Yang L, Wang L, Yang Z, Jin H, Zou Q, Zhan Q, et al. Up-regulation of EMT-related gene VCAN by NPM1 mutant-driven TGF-β/cPML signalling promotes leukemia cell invasion. J Cancer. 2019;10:6570–83. 31777586 10.7150/jca.30223 PMC6856892

[42] Stavropoulou V, Kaspar S, Brault L, Sanders MA, Juge S, Morettini S, et al. MLL-AF9 expression in hematopoietic stem cells drives a highly invasive AML expressing EMT-related genes linked to poor outcome. Cancer Cell. 2016;30:43–58. 27344946 10.1016/j.ccell.2016.05.011

[43] Tang DE, Dai Y, Xu Y, Lin LW, Liu DZ, Hong XP, et al. The ubiquitinase ZFP91 promotes tumor cell survival and confers chemoresistance through FOXA1 destabilization. Carcinogenesis. 2020;41:56–66. 31046116 10.1093/carcin/bgz085

[44] Rao SS, Huntley MH, Durand NC, Stamenova EK, Bochkov ID, Robinson JT, et al. A 3D map of the human genome at kilobase resolution reveals principles of chromatin looping. Cell. 2014;159:1665–80. 25497547 10.1016/j.cell.2014.11.021 PMC5635824

[45] Wang Y, Song F, Zhang B, Zhang L, Xu J, Kuang D, et al. The 3D Genome Browser: a web-based browser for visualizing 3D genome organization and long-range chromatin interactions. Genome Biol. 2018;19:151. 30286773 10.1186/s13059-018-1519-9 PMC6172833

[46] Ng HL, Robinson ME, May PC, Innes AJ, Hiemeyer C, Feldhahn N. Promoter-centred chromatin interactions associated with EVI1 expression in EVI1+3q- myeloid leukaemia cells. Br J Haematol. 2024;204:945–58. 38296260 10.1111/bjh.19322

[47] Ayoub E, Wilson MP, McGrath KE, Li AJ, Frisch BJ, Palis J, et al. EVI1 overexpression reprograms hematopoiesis via upregulation of Spi1 transcription. Nat Commun. 2018;9:4239. 30315161 10.1038/s41467-018-06208-y PMC6185954

[48] Schmoellerl J, Barbosa IAM, Minnich M, Andersch F, Smeenk L, Havermans M. et al. EVI1 drives leukemogenesis through aberrant ERG activation. Blood. 2022;141:453–66. 10.1182/blood.2022016592 36095844

[49] Elster D, Tollot M, Schlegelmilch K, Ori A, Rosenwald A, Sahai E, et al. TRPS1 shapes YAP/TEAD-dependent transcription in breast cancer cells. Nat Commun. 2018;9:3115. 30082728 10.1038/s41467-018-05370-7 PMC6079100

[50] Yamamoto A, Kurata M, Onishi I, Sugita K, Matsumura M, Ishibashi S, et al. CRISPR screening identifies M1AP as a new MYC regulator with a promoter-reporter system. PeerJ. 2020;8:e9046. 32411526 10.7717/peerj.9046 PMC7210806

[51] Zhang H, Zhang Y, Zhou X, Wright S, Hyle J, Zhao L. et al. Functional interrogation of HOXA9 regulome in MLLr leukemia via reporter-based CRISPR/Cas9 screen. eLife. 2020;9:453–66. 10.7554/eLife.57858 PMC7599066 33001025

[52] Spisak S, Chen D, Likasitwatanakul P, Doan P, Li Z, Bala P, et al. Identifying regulators of aberrant stem cell and differentiation activity in colorectal cancer using a dual endogenous reporter system. Nat Commun. 2024;15:2230. 38472198 10.1038/s41467-024-46285-w PMC10933491

[53] Hanafi M, Chen X, Neamati N. Discovery of a napabucasin PROTAC as an effective degrader of the E3 ligase ZFP91. J Med Chem. 2021;64:1626–48. 33506674 10.1021/acs.jmedchem.0c01897 PMC8083113

[54] An J, Ponthier CM, Sack R, Seebacher J, Stadler MB, Donovan KA, et al. pSILAC mass spectrometry reveals ZFP91 as IMiD-dependent substrate of the CRL4. Nat Commun. 2017;8:15398. 28530236 10.1038/ncomms15398 PMC5458144

[55] Chen D, Wang Y, Lu R, Jiang X, Chen X, Meng N, et al. E3 ligase ZFP91 inhibits hepatocellular carcinoma metabolism reprogramming by regulating PKM splicing. Theranostics. 2020;10:8558–72. 32754263 10.7150/thno.44873 PMC7392027

[56] Zhu Y, Zhang J, Ye Z, Peng P, Lan X, Xie L, et al. Evi-1 influence on clinical progress of colorectal cancer patients. Ann Clin Lab Sci. 2020;50:354–60. 32581025

[57] Kim HR, Yim J, Yoo HB, Lee SE, Oh S, Jung S, et al. EVI1 activates tumor-promoting transcriptional enhancers in pancreatic cancer. NAR Cancer. 2021;3:zcab023. 34316710 10.1093/narcan/zcab023 PMC8210884

[58] Yasui K, Konishi C, Gen Y, Endo M, Dohi O, Tomie A, et al. EVI1, a target gene for amplification at 3q26, antagonizes transforming growth factor-β-mediated growth inhibition in hepatocellular carcinoma. Cancer Sci. 2015;106:929–37. 25959919 10.1111/cas.12694 PMC4520646

