# Supplementary material for: Genome-wide screening identifies ZFP91 as a key regulator of EVI1 in myeloid leukemia
Source: Oncogene. 2026 Apr 25;45(23):2237–48. doi: 10.1038/s41388-026-03727-7 (PMC13246443; doi:10.1038/s41388-026-03727-7)
Supplement: Supplementary file 1 — Supplemental Document [file 41388_2026_3727_MOESM1_ESM.docx]

**SUPPLEMENTARY INFORMATION**

Supplementary Information contains the following:

- Supplementary Methods
- Supplementary Figure/Table Legends
- Supplementary Figures 1–3
- Supplementary Tables 1–2 (provided in Excel format).

**SUPPLEMENTARY METHODS**

**Data availability**

The RNA-seq, ChIP-seq, and ATAC-seq generated in this study are available in NCBI Sequence Read Archive (SRA) (RRID:SCR_004891) at PRJDB19903, and the corresponding processed data are available in Genomic Expression Archive (GEA) at E-GEAD-891, E-GEAD-900, and E-GEAD-893. The ChIP-seq for CTCF or MECOM in K562 cells and for ZFP91 in HepG2 cells were obtained from ENCODE (RRID:SCR_015482): ENCFF268ZPN for CTCF in K562 cells; ENCFF664MHN, ENCFF411DHX, ENCFF291RWA, and ENCFF521ELI for MECOM in K562 cells; ENCFF884FER and ENCFF429XRR for control in K562 cells; ENCFF209OOA and ENCFF852OEO for ZFP91 in HepG2 cells; ENCFF705PQR for control in HepG2 cells. The Cancer Genome Atlas (TCGA) (RRID:SCR_003193)[1] and Databases at The Oregon Health & Science University (OHSU) (RRID:SCR_009665)[2] were obtained from cBioPortal (RRID:SCR_014555)[3-5]. Topologically associated domain data in K562 cells were obtained from 3D Genome Browser[6].

**Cell Lines**

K562 (JCRB, RRID:CVCL_0004), HEL (JCRB, RRID:CVCL_0001), CMK-11-5 (JCRB, CVCL_0217), F-36P (RIKEN, RRID:CVCL_2037) and THP-1 (JCRB, RRID:CVCL_0006) cells were cultured in RPMI-1640 (nacalai tesque) supplemented with 10% fetal bovine serum (FBS). Plat-A (Kitamura et al., RRID:CVCL_B489) and Lenti-X™ 293T (Takara Bio Cat#632180, RRID:CVCL_4401) cells were cultured in DMEM (High Glucose) (nacalai tesque) supplemented with 10% FBS, and trypsinized at passage. All cell lines were cultured with 100 units/mL penicillin and 100 μg/mL streptomycin in a 5% CO_2_ incubator at 37°C. Cells were counted using a hemocytometer after staining with trypan blue.

**Plasmid Construction**

Plasmids or PCR products were purified using NucleoSpin^®^ Gel and PCR clean-up (Takara Bio, Cat#740609). Digested plasmids were ligated with oligos using Ligation High Ver.2 (TOYOBO, Cat#LGK-201), and transformed into *E. coli* DH5α Competent Cells (Takara Bio, Cat#9057) by heat shock, followed by the verification with colony PCR using KOD FX Neo (TOYOBO, Cat#KFX-201) and Sanger sequencing (Fasmac, Europhins Genomics). Plasmid DNA was extracted from the competent cells using NucleoBond^®^ Xtra Midi (Takara Bio, Cat#740410) or NucleoSpin^®^ Plasmid EasyPure (Takara Bio, Cat#740727). DNA concentrations were measured using NanoDrop One (Thermo Fisher Scientific).

The annealed oligos were cloned into pSIREN-RetroQ (Takara Bio, Cat#631526) for EVI1 knockdown, or lentiGuide-puro (Feng Zhang, RRID:Addgene_52963) for lentiviral knockout. For ZFP91 overexpression, RNA was extracted from K562 cells using NucleoSpin^®^ RNA (Takara Bio, Cat#740955), and complementary DNA (cDNA) was synthesized using ReverTra Ace^®^ qPCR RT Master Mix (TOYOBO, Cat#FSQ-201). ZFP91 coding sequence (CCDS31553.1), amplified by PCR from the cDNA using KOD One^®^ PCR Master Mix (TOYOBO, Cat#KMM-101) with the forward primer included Kozak and 1×FLAG tag sequence, was first cloned into pENTR1A and then subcloned into pMYs-IRES-GFP. ZFP91 construct on pMYs-IRES-GFP carrying a silent mutation that disrupts the protospacer adjacent motif (PAM) sequence to evade recognition by sgZFP91#3 was first generated using KOD -Plus- Mutagenesis Kit (TOYOBO, Cat#SMK-101) (with KOD One^®^ PCR Master Mix substituted as the polymerase). This sgRNA-resistant construct then served as the template for introducing cysteine-to-alanine mutations in the zinc finger (ZnF) domains. Mutations in ZnF1–4 were introduced using the KOD -Plus- Mutagenesis Kit (with KOD One^®^ PCR Master Mix substituted as the polymerase), whereas the mutation in ZnF5 was generated using NEBuilder^®^ HiFi DNA Assembly Master Mix (New England Biolabs, Cat#E2621) due to technical considerations.

For luciferase promoter assay, the *EVI1* promoter sequence was amplified by PCR from HEL genomic DNA and cloned into pGL4.10 (Promega, Cat#E6651). The ZFP91 motifs were converted to random sequences by Gibson assembly using NEBuilder^®^ HiFi DNA Assembly Master Mix.

**Establishment of GFP Knock-in Cell Lines**

The repair template was prepared using Gibson assembly as previously described[7]. The homology arms, GSG-P2A, and EGFP sequences were PCR amplified from K562 genomic DNA, pMX-GSG-P2A-ΔNGFR[8] and pMYs-IRES-GFP, respectively. The single-stranded oligodeoxynucleotides repair template generated using Guide-it™ Long ssDNA Production System (Takara Bio, Cat#632644) were co-transfected into K562 cells with Alt-R^®^ S.p. Cas9 Nuclease V3 (Integrated DNA Technologies, Cat#1081058), Alt-R^®^ tracrRNA (Integrated DNA Technologies, Cat#1072532), and Alt-R^®^ crRNA (Integrated DNA Technologies) targeting the sequence spanning the *EVI1* stop codon by electroporation using NEPA21 electroporator (Nepa Gene). After a period of culture, GFP-positive cells were single-cell sorted using FACSAria™ Ⅱ (BD Biosciences). Successful knock-in was confirmed by PCR and Sanger sequencing of the genome, followed by flowcytometric analysis using FACSCelesta™ flow cytometer (BD Biosciences).

**Retroviral or Lentiviral Transduction**

Retroviral vectors were transfected into Plat-A cells using polyethyleneimine (PEI). After 48 hours, the viral supernatant was collected, passed through a 0.45 µm Millex^®^ filter (Millipore, Cat#SLHPR33RS), and centrifuged onto RetroNectin^®^ (Takara Bio, Cat#T100B) coated plates at 2,000 rpm for 2 hours. After virus removal, cells were incubated on the plate for 24-48 hours. Infected cells were selected by puromycin or GFP sorting using FACSAria™ Ⅲ.

Lentiviral vectors were co-transfected with psPAX2 and pMD2.G (Didier Trono, RRID:Addgene_12260 and RRID:Addgene_12259) into Lenti-X™ 293T cells using PEI. Viral fluids were harvested in the same manner as for retroviruses. The virus solution was ultracentrifuged at 100,000×g for 2 hours, or alternatively, mixed with polyethylene glycol (PEG), left overnight, and then centrifuged at 2,000 rpm for 1 hour, all at 4°C. The virus pellets were resuspended in PBS or medium to 200× concentration and stored at -80°C. Lentiviral transduction was performed using polybrene or RetroNectin^®^ as previously described[9, 10].

**Western Blot**

Cells were lysed in lysis buffer (10 mM Tris-HCl, 1 mM EDTA, 150 mM NaCl, 1% NP-40, 12.5 mM β-glycerophosphate, 1 mM phenylmethylsulfonyl fluoride (PMSF), 1 mM sodium orthovanadate) containing cOmpleteTM protease inhibitor cocktail (PI) (Roche, Cat#5056489001). Protein concentrations were determined by the Bradford protein assay (Bio-Rad, Cat#500-0006). Samples were denatured by boiling at 95°C for 5 minutes with 4×sample buffer (200 mM Tris-HCl, 6.4% sodium dodecyl sulfate (SDS), 32% glycerol, 0.04% bromophenol blue and 20% 2-mercaptoethanol (2-ME)) and analyzed by SDS-polyacrylamide gel electrophoresis. Proteins were detected with the following primary antibodies: Cas9 (*S. pyogenes*) (7A9-3A3) Mouse mAb (Cell Signaling Technology, Cat#14697, RRID:AB_2750916), Evi-1 (C50E12) Rabbit mAb (Cell Signaling Technology, Cat#2593, RRID:AB_2184098), ZFP91 Polyclonal Antibody (Bethyl, Cat#A303-245A, RRID:AB_10953803), or β-Actin Antibody (Cell Signaling Technology, Cat#4967, RRID:AB_330288). After the incubation with Anti-mouse IgG, HRP-linked Antibody (Cell Signaling Technology, Cat#7076, RRID:AB_330924) or Anti-rabbit IgG, HRP-linked Antibody (Cell Signaling Technology, Cat#7074, RRID:AB_2099233), proteins were reacted with ImmunoStar LD (Fujifilm Wako, Cat#296-69901) or Chemi-Lumi One Super (nacalai tesque, Cat#02230-30), and visualized using ImageQuant™ LAS 4000 mini CCD imager (Cytiva). After incubation in stripping buffer (62.5 mM Tris-HCl, 2% SDS, 0.8% 2-ME) at 50°C for 30 minutes, β-actin was detected in the same manner using β-Actin Antibody. Antibodies were diluted to 1:1000 - 1:2000 with 5% skim milk or 5% bovine serum albumin in TNT buffer (10 mM Tris-HCl, 150 mM NaCl, 0.1% Tween-20) or with Can Get Signal^®^ Immunoreaction Enhancer Solution (TOYOBO, Cat#NKB-101).

**Quantitative Reverse Transcription PCR (qRT-PCR)**

RNA and cDNA was prepared as described above, and qRT-PCR was performed using THUNDERBIRD^®^ SYBR™ qPCR Mix (TOYOBO, Cat#QPS-201) or THUNDERBIRD^®^ Next SYBR™ qPCR Mix (TOYOBO, Cat#QPX-201) on QuantStudio 5 or 6 (Thermo Fisher Scientific). Reference genes used for the initial validation study in K562, HEL and CMK-11-5 cells were selected by GeNorm[11] and BestKeeper[12] algorithms on R using ctrlGene package (DOI: [10.32614/CRAN.package.ctrlGene](https://doi.org/10.32614/CRAN.package.ctrlGene)) from the following genes: *ACTB, B2M, GAPDH, GUSB, HPRT1, PGK1, PPIA, RPL13A, SDHA, TBP, TFRC, UBC, TWHAZ*, and 18s rRNA. For the subsequent experiments, we used *PGK1* and *YWHAZ* as reference genes, because they were commonly high ranking in the three cell lines. Relative expression levels of target genes were determined by dividing by geometric mean of the expression levels of the two reference genes. Statistical analysis was performed on log-transformed relative expression levels.

**Intracellular Flow Cytometry**

Cells were fixed with 4% formaldehyde at room temperature for 15 minutes, incubated in methanol at -20°C, and stained with Evi-1 (C50E12) Rabbit mAb (Cell Signaling Technology Cat#2593, RRID:AB_2184098) followed by Alexa Fluor^®^ 647 Donkey anti-rabbit IgG (min. x-reactivity) (BioLegend, Cat#406414, RRID:AB_2563202). The fluorescence intensity was analyzed on FACSCelesta™ flow cytometer. Statistical analysis was performed on log-transformed median fluorescence intensity (MFI) for each gene.

**Luciferase Promoter Assay**

Approximately 2.5×10^4^ control K562 cells or 4×10^4^ ZFP91-knockout K562 cells were seeded on a 96-well plate 24 hours prior to transfection. Cells were co-transfected with 100 ng of pGL4.10 and 5 ng of pRL-TK (Promega, Cat#E2241) using 0.1 µL of TransIT-2020 Transfection Reagent (Takara Bio, Cat#MIR5410). Three days after transfection, Dual-Glo^®^ Luciferase Assay System (Promega, Cat#E2920) was used to measure firefly luciferase luminescence, followed by *Renilla* luciferase luminescence, using ARVO™ MX plate reader (PerkinElmer).

**Pooled CRISPR Screening**

Human GeCKOv2 CRISPR knockout pooled library and Cas9-expressing lentiviral vector were provided from Feng Zhang (Addgene #1000000049)[13]. CRISPR screening was performed as previously reported[9]. Briefly, 2.7×10^8^ K562-EVI1-GFP-Cas9 cells were infected with each half library lentivirus at an MOI of 0.3–0.4, selected by puromycin, and harvested 6–7 days after transduction. Cells in top 20% and bottom 20% of GFP fluorescence intensity were sorted using FACSAria™ Ⅱ & Ⅲ (BD Bioscience). The coverage of cell counts to the number of sgRNAs was confirmed to be greater than 250-fold. Genomic DNA was extracted using NucleoSpin^®^ Blood L (TakaraBio, Cat#740954), and sgRNA cassettes were amplified by PCR using NEBNext Ultra II Q5 Master Mix (New England Biolabs, Cat#M0544). Purified PCR products were analyzed by Illumina HiSeq 2×150 bp sequencing, generating 30–40 million paired-end reads per sample (Azenta Life Sciencies).

**RNA-seq**

RNA was extracted using NucleoSpin^®^ RNA. The library was prepared by NEBNext^®^ Poly(A) mRNA Magnetic Isolation Module (New England Biolabs, Cat#E7490), followed by NEBNext^®^ Ultra™ II Directional RNA Library Prep Kit for Illumina^®^ (New England Biolabs, Cat#E7760). Sequencing was performed on Illumina NovaSeq 6000 with 2×150 bp read length, generating 20–30 million paired-end reads per sample (Rhelixa).

**ChIP-qPCR and ChIP-seq**

ChIP experiment was carried out as previously described[14]. Briefly, cells were fixed with formaldehyde, treated with micrococcal nuclease (MNase), and sonicated using Branson digital Sonifier^®^ 250 (Emerson). The samples were incubated with the following antibodies cross-linked to Dynabeads™ Protein G (Invitrogen, Cat#10004D): ZFP91 Polyclonal Antibody (Bethyl, Cat#A303-245A, RRID:AB_10953803), Tri-Methyl-Histone H3 (Lys4) (C42D8) Rabbit mAb (Cell Signaling Technology, Cat#9751, RRID:AB_2616028), Anti-Histone H3 (acetyl K27) antibody - ChIP Grade (Abcam, Cat#ab4729, RRID:AB_2118291), and Normal Rabbit IgG (Cell Signaling Technology, Cat#2729, RRID:AB_1031062). After reverse cross-linking by boiling with SDS, the samples were treated with RNaseA, followed by proteinase K. DNA was extracted using NucleoSpin^®^ Gel and PCR clean-up. ChIP-qPCR was performed using THUNDERBIRD^®^ SYBR™ qPCR Mix on QuantStudio 6. Library for next generation sequencing was prepared using NEBNext^®^ Ultra™ II DNA Library Prep Kit for Illumina^®^ (New England Biolabs, Cat#E7645) and analyzed by Illumina NextSeq 2000 sequencing with 2×36 bp read length, generating 30–40 million paired-end reads per sample (Katsuhiko Shirahige Lab.).

**ATAC-seq**

ATAC-seq library was prepared as previously described[15]. Briefly, 5×10^5^ live cells were collected by FACSAria™ Ⅲ using 7-AAD staining (BioLegend, Cat#420404). Cells were lysed, and treated with Tn5 transposase (Diagenode, Cat#C01070012 and Cat#C01019043). DNA was extracted using NucleoSpin^®^ Gel and PCR clean-up, and amplified by PCR using NEBNext Ultra II Q5. PCR cycle number was determined using NEBNext^®^ Library Quant Kit for Illumina^®^(New England Biolabs, Cat#E7630). The PCR products were purified using Agencourt^®^ AMPure^®^ XP (Beckman Coulter, Cat#A63880), and analyzed by Illumina NextSeq 2000 sequencing with 2×36 bp read length, generating 60 million paired-end reads per sample (Katsuhiko Shirahige Lab.).

**Data Analysis**

For CRISPR screening, reads 1 were trimmed using Cutadapt (RRID:SCR_011841)[16] v4.9 with the options -a
GCTTTATATATCTTGTGGAAAGGACGAAACACCG...GTTTTAGAGCTAGAAATAGCAAGTTAAAATAAGGCTAGTCCGTTATCAACTTGAAAAAGTGGCACCGAGTCGG -O 34 -m 20 -M 20 --trimmed-only. Reads in each half library were counted and normalized separately using MAGeCK (RRID:SCR_025016)[17] v0.5.9.5 count command with the default options, and integrated by the cat command. Normalized read counts were statistically compared between GFP^Low^ and GFP^High^ using the MAGeCK test command with the option --norm-method control.

For RNA-seq, paired-end reads were trimmed using Fastp (RRID:SCR_016962)[18] v0.23.4 in paired-end mode with the option --detect_adapter_for_pe. The trimmed reads were mapped to the primary assembled human genome GRCh38 without masking from Ensembl Release 110 using STAR (RRID:SCR_004463)[19] v2.7.11a, and counted using RSEM (RRID:SCR_000262)[20] v1.3.1, both with the default options. Differentially expressed genes were analyzed using DESeq2 (RRID:SCR_015687)[21] v1.44.0, and GSEA and GO enrichment analysis were performed using clusterProfiler (RRID:SCR_016884)[22] v4.12.6, both on R 4.4.1. Bubble plots were generated using DOSE[23] v4.0.0.

For ChIP-seq and ATAC-seq, Bowtie2-index of the human genome hg38 was downloaded using Refgenie (RRID:SCR_017574)[24, 25] v0.12.1. Sequenced reads were used directly for mapping for ChIP-seq, while for ATAC-seq they were trimmed in advance using Fastp v0.23.4 in paired-end mode with the option -a CTGTCTCTTATACACATCT. Reads were mapped to hg38 using Bowtie 2 (RRID:SCR_016368)[26] v2.5.2 with the default options for ChIP-seq or with the options --very-sensitive --no-mixed --no-discordant -X 2000 for ATAC-seq. Concordantly and uniquely mapped reads were retained using SAMtools (RRID:SCR_002105)[27] v1.20 view command with the options -f 0x2 -q 20 for paired-end data or with the options -F 0x4 -q 20 for single-end data. Reads mapped to the mitochondrial genome were removed for ATAC-seq using the grep command with the -v chrM option. Duplicated reads were removed using Picard (RRID:SCR_006525) v3.2.0 MarkDuplicates. For ATAC-seq, filtered paired-end bam files were rewritten to single-end bam files by changing flag 99 or 163 to flag 0, and flag 83 or 147 to flag 16, and renaming reads to avoid duplication. This modification allowed the inclusion of read 2 information for peak calling in ATAC-seq. Peak calling for each replicate was performed using the MACS3 (RRID:SCR_013291)[28] v3.0.1 callpeak command with the options -g hs -B -q 0.01 for ChIP-seq, or with the options -f BAM --nomodel --shift -36 --extsize 72 -g hs -B -q 0.01 for ATAC-seq. For peak visualization on Integrative Genomics Viewer (IGV) (RRID:SCR_011793)[29], the MACS3 callpeak command with merged replicates for each biological condition was executed, using the --SPMR option. This was followed by the MACS3 bdgcmp command with the -m FE option for ChIP-seq, while for ATAC-seq, the output “_treat_pileup.bdg” files from the MACS3 callpeak command were directly used for visualization. Peak regions for transcription factors were determined as the region common to all the biological replicates using the BEDtools (RRID:SCR_006646)[30] v2.31.1 intersect command. ENCODE blacklist regions[31] were removed for the subsequent analyses. Statistical quantitative peaks were compared using MAnorm2[32] v1.2.2. The bed/csv files required for MAnorm2 were generated using the MAnorm2_utils v1.0.0 profile_bins command with the option --paired for ChIP-seq or with the option --shiftsize 0 for ATAC-seq. Motif analysis for ChIP-seq was performed using HOMER (RRID:SCR_010881)[33] v4.10.4. Footprinting analysis for ATAC-seq was performed using TOBIAS[34] 0.13.3 with HOCOMOCO v12 (RRID:SCR_005409)[35] and JASPAR 2024 (RRID:SCR_003030)[36] motif databases.

For the comparative analysis of differential binding scores between regions inside and outside EVI1-ChIP peaks, we followed these steps: First, for each less-occupied motif in ZFP91 knockout, we extracted regions expected to be bound by transcription factors in at least one of the conditions (NT or KO) from TOBIAS BINDetect output "_overview.txt" files. Next, we used the BEDtools intersect command to divide these bound regions into two groups for each motif: those inside EVI1-ChIP peaks and those outside EVI1-ChIP peaks. Finally, the mean log2 fold-change (KO/NT) of the binding score for each motif was calculated separately for the regions inside EVI1-ChIP peaks and those outside, and statistically analyzed by paired *t* test.

**Statistical Analysis**

Geometric means and geometric standard deviations were plotted for relative mRNA expression levels, median fluorescence intensity, luminescence, %input in ChIP-qPCR, and relative cell counts. Statistical analyses for them were performed on log-transformed values to stabilize variance and improve the approximation to normality.

The qRT-PCR and the intracellular flow cytometry performed on top-ranked genes from the CRISPR screening were exploratory validation experiments. Therefore, statistical comparisons were conducted using Welch’s analysis of variance (ANOVA), followed by unpaired Welch’s *t* test for pairwise comparisons against the NT group without correction for multiple comparisons (Fig. 1E–H and Supplementary Fig. 1D).

To assess differences in mRNA expression (qRT-PCR) or %input (ChIP-qPCR) between NT and KO groups, two-way repeated measures ANOVA was performed with Geisser-Greenhouse correction (Fig. 2D, E, and 4H). Biological replicates (NT: n=2; KO: n=3) and technical replicates (n=3) were incorporated within a single model.

Changes in *EVI1* or *ZFP91* mRNA expression after wild-type or mutant ZFP91 overexpression were assessed using relative expression ratios normalized to the control (mock for Supplementary Fig. 1E, F; NT2 for Supplementary Fig. 2C, D). Statistical comparisons were performed using paired *t* test (Supplementary Fig. 1E, F and 2D). If multiple test samples were compared against a single control, adjusted p-values were calculated using the Holm-Bonferroni method (Supplementary Fig. 2D). When the compared control group was not used for normalization, Welch’s ANOVA followed by Dunnett’s test was applied to compare multiple test conditions against a single control (Supplementary Fig. 2C).

In promoter assay, firefly luciferase luminescence was normalized with *Renilla* luciferase luminescence. One-way repeated measures ANOVA with Geisser-Greenhouse correction was first performed separately for each group with p-values adjusted for multiple testing using the Holm-Bonferroni method. Dunnett’s test was then applied for post hoc comparisons in groups showing significant ANOVA result to compare multiple test conditions against a single control (e.g. NT vs. KO2/KO3, or original vs. Δmotif-1/-2) (Fig. 4D, G).

For the scatterplot comparing log₂ fold-changes after ZFP91 or EVI1 knockout, the correlation coefficient and its p-value were computed using two-sided Pearson correlation test (Fig. 3B).

Paired *t* tests were used for matched samples (Fig. 5E), and Welch’s *t* tests for independent samples (Fig. 5I).

Cell growth was analyzed using a linear mixed-effects model with log-transformed relative cell counts as the response variable (Fig. 5G, H). Day and the interaction between day and group were included as fixed effects, with day treated as a continuous variable. Experimental batch was included as a random intercept to account for variability between experiments. The group × day interaction was used to assess differences in proliferation rates between groups (Fig, 5G, H).

For CRISPR screening, RNA-seq, ChIP-seq, and ATAC-seq, data were analyzed using the packages described in the Data Analysis section. Linear mixed-effects models were fitted in R 4.4.2 using lme4 (RRID:SCR_015654) and lmerTest (RRID:SCR_015656) packages. All other statistical analyses were performed using GraphPad Prism 10 (RRID:SCR_002798).

**Reference**

1 Cancer Genome Atlas Research N, Ley TJ, Miller C, Ding L, Raphael BJ, Mungall AJ *et al*. Genomic and epigenomic landscapes of adult de novo acute myeloid leukemia. *N Engl J Med* 2013; 368: 2059-2074.

2 Bottomly D, Long N, Schultz AR, Kurtz SE, Tognon CE, Johnson K *et al*. Integrative analysis of drug response and clinical outcome in acute myeloid leukemia. *Cancer Cell* 2022; 40: 850-864.e859.

3 Cerami E, Gao J, Dogrusoz U, Gross BE, Sumer SO, Aksoy BA *et al*. The cBio cancer genomics portal: an open platform for exploring multidimensional cancer genomics data. *Cancer Discov* 2012; 2: 401-404.

4 Gao J, Aksoy BA, Dogrusoz U, Dresdner G, Gross B, Sumer SO *et al*. Integrative analysis of complex cancer genomics and clinical profiles using the cBioPortal. *Sci Signal* 2013; 6: pl1.

5 de Bruijn I, Kundra R, Mastrogiacomo B, Tran TN, Sikina L, Mazor T *et al*. Analysis and Visualization of Longitudinal Genomic and Clinical Data from the AACR Project GENIE Biopharma Collaborative in cBioPortal. *Cancer Res* 2023; 83: 3861-3867.

6 Wang Y, Song F, Zhang B, Zhang L, Xu J, Kuang D *et al*. The 3D Genome Browser: a web-based browser for visualizing 3D genome organization and long-range chromatin interactions. *Genome Biol* 2018; 19: 151.

7 Smeenk L, Ottema S, Mulet-Lazaro R, Ebert A, Havermans M, Varea AA *et al*. Selective Requirement of MYB for Oncogenic Hyperactivation of a Translocated Enhancer in Leukemia. *Cancer Discov* 2021; 11: 2868-2883.

8 Kagoya Y, Tanaka S, Guo T, Anczurowski M, Wang CH, Saso K *et al*. A novel chimeric antigen receptor containing a JAK-STAT signaling domain mediates superior antitumor effects. *Nat Med* 2018; 24: 352-359.

9 Joung J, Konermann S, Gootenberg JS, Abudayyeh OO, Platt RJ, Brigham MD *et al*. Genome-scale CRISPR-Cas9 knockout and transcriptional activation screening. *Nat Protoc* 2017; 12: 828-863.

10 Schimmer AD, Singh RP, Seneviratne AK, Thomas GE, MacLean N, Hurren R. Transduction of Primary AML Cells with Lentiviral Vector for. *STAR Protoc* 2020; 1: 100163.

11 Vandesompele J, De Preter K, Pattyn F, Poppe B, Van Roy N, De Paepe A *et al*. Accurate normalization of real-time quantitative RT-PCR data by geometric averaging of multiple internal control genes. *Genome Biol* 2002; 3: RESEARCH0034.

12 Pfaffl MW, Tichopad A, Prgomet C, Neuvians TP. Determination of stable housekeeping genes, differentially regulated target genes and sample integrity: BestKeeper--Excel-based tool using pair-wise correlations. *Biotechnol Lett* 2004; 26: 509-515.

13 Sanjana NE, Shalem O, Zhang F. Improved vectors and genome-wide libraries for CRISPR screening. *Nat Methods* 2014; 11: 783-784.

14 Yoshimi A, Goyama S, Watanabe-Okochi N, Yoshiki Y, Nannya Y, Nitta E *et al*. Evi1 represses PTEN expression and activates PI3K/AKT/mTOR via interactions with polycomb proteins. *Blood* 2011; 117: 3617-3628.

15 Grandi FC, Modi H, Kampman L, Corces MR. Chromatin accessibility profiling by ATAC-seq. *Nat Protoc* 2022; 17: 1518-1552.

16 Martin M. Cutadapt removes adapter sequences from high-throughput sequencing reads. *2011* (next generation sequencing; small RNA; microRNA; adapter removal) 2011; 17: 3.

17 Li W, Xu H, Xiao T, Cong L, Love MI, Zhang F *et al*. MAGeCK enables robust identification of essential genes from genome-scale CRISPR/Cas9 knockout screens. *Genome Biol* 2014; 15: 554.

18 Chen S. Ultrafast one-pass FASTQ data preprocessing, quality control, and deduplication using fastp. *iMeta* 2023; 2: e107.

19 Dobin A, Davis CA, Schlesinger F, Drenkow J, Zaleski C, Jha S *et al*. STAR: ultrafast universal RNA-seq aligner. *Bioinformatics* 2013; 29: 15-21.

20 Li B, Dewey CN. RSEM: accurate transcript quantification from RNA-Seq data with or without a reference genome. *BMC Bioinformatics* 2011; 12: 323.

21 Love MI, Huber W, Anders S. Moderated estimation of fold change and dispersion for RNA-seq data with DESeq2. *Genome Biol* 2014; 15: 550.

22 Wu T, Hu E, Xu S, Chen M, Guo P, Dai Z *et al*. clusterProfiler 4.0: A universal enrichment tool for interpreting omics data. *Innovation (Camb)* 2021; 2: 100141.

23 Yu G, Wang LG, Yan GR, He QY. DOSE: an R/Bioconductor package for disease ontology semantic and enrichment analysis. *Bioinformatics* 2015; 31: 608-609.

24 Stolarczyk M, Reuter VP, Smith JP, Magee NE, Sheffield NC. Refgenie: a reference genome resource manager. *Gigascience* 2020; 9.

25 Stolarczyk M, Xue B, Sheffield NC. Identity and compatibility of reference genome resources. *NAR Genom Bioinform* 2021; 3: lqab036.

26 Langmead B, Salzberg SL. Fast gapped-read alignment with Bowtie 2. *Nat Methods* 2012; 9: 357-359.

27 Danecek P, Bonfield JK, Liddle J, Marshall J, Ohan V, Pollard MO *et al*. Twelve years of SAMtools and BCFtools. *Gigascience* 2021; 10.

28 Zhang Y, Liu T, Meyer CA, Eeckhoute J, Johnson DS, Bernstein BE *et al*. Model-based analysis of ChIP-Seq (MACS). *Genome Biol* 2008; 9: R137.

29 Robinson JT, Thorvaldsdóttir H, Winckler W, Guttman M, Lander ES, Getz G *et al*. Integrative genomics viewer. *Nat Biotechnol* 2011; 29: 24-26.

30 Quinlan AR, Hall IM. BEDTools: a flexible suite of utilities for comparing genomic features. *Bioinformatics* 2010; 26: 841-842.

31 Amemiya HM, Kundaje A, Boyle AP. The ENCODE Blacklist: Identification of Problematic Regions of the Genome. *Sci Rep* 2019; 9: 9354.

32 Tu S, Li M, Chen H, Tan F, Xu J, Waxman DJ *et al*. MAnorm2 for quantitatively comparing groups of ChIP-seq samples. *Genome Res* 2021; 31: 131-145.

33 Heinz S, Benner C, Spann N, Bertolino E, Lin YC, Laslo P *et al*. Simple combinations of lineage-determining transcription factors prime cis-regulatory elements required for macrophage and B cell identities. *Mol Cell* 2010; 38: 576-589.

34 Bentsen M, Goymann P, Schultheis H, Klee K, Petrova A, Wiegandt R *et al*. ATAC-seq footprinting unravels kinetics of transcription factor binding during zygotic genome activation. *Nat Commun* 2020; 11: 4267.

35 Vorontsov IE, Eliseeva IA, Zinkevich A, Nikonov M, Abramov S, Boytsov A *et al*. HOCOMOCO in 2024: a rebuild of the curated collection of binding models for human and mouse transcription factors. *Nucleic Acids Res* 2024; 52: D154-D163.

36 Rauluseviciute I, Riudavets-Puig R, Blanc-Mathieu R, Castro-Mondragon JA, Ferenc K, Kumar V *et al*. JASPAR 2024: 20th anniversary of the open-access database of transcription factor binding profiles. *Nucleic Acids Res* 2024; 52: D174-D182.

**SUPPLEMENTARY FIGURE/TABLE LEGENDS**

**Supplementary Fig. 1: Complementary results of the CRISPR screening and the wild-type ZFP91 overexpression experiment, related to Fig. 1 and Fig. 2.**

**A** Top ranked genes enriched in GFP^Low^ fraction with FDR<25%. The “num” in the second column represents the number of sgRNA sequences in each gene or micro-RNA in the whole library. The “score” in the third column represents robust ranking aggregation (RRA) score for each gene. The “goodsgrna” in the seventh column represents the number of sgRNAs with FDR<25% for each gene. The library contains 6 sgRNAs for each protein-coding gene and 4 sgRNAs for each micro-RNA. The “lfc” in the last column represents the median log2(fold-change) of sgRNAs for each gene or micro-RNA. Shading row indicates genes used for individual validation. **B** Top ranked genes enriched in GFP^High^ fraction with FDR<25%. **C** A scatter plot showing genes enriched in GFP^High^ population from the CRISPR screen. The horizontal axis indicates median log2(fold-change) of sgRNAs for each gene relative to GFP^Low^ fraction, while the vertical axis represents log2(RRA score). Each dot corresponds to a gene, with purple dots indicating those with FDR<1%. **D** GFP fluorescence in K562-EVI1-GFP-Cas9 cells after transduction of sgRNAs targeting the candidate genes. GFP intensity were assessed 4 days post-transduction. **E, F** *EVI1* and *ZFP91* mRNA expression after ZFP91 overexpression in K562, HEL, CMK-11-5, F-36P and THP-1 cells. Relative expressions compared to mock control were normalized to *PGK1* and *YWHAZ*.
All bar graphs depict geometric means ± geometric standard deviations. Statistical significance was determined by Welch’s analysis of variance (ANOVA) followed by unpaired Welch’s *t* tests on log-transformed values for (D), or by paired *t* tests on log-transformed expression ratios for (E, F), with *p<0.05, **p<0.01, ***p<0.001, ****p<0.0001.

**Supplementary Fig. 2: Commonly altered pathways identified by RNA-seq in ZFP91- and EVI1-knockout K562 cells, and effects of zinc finger mutant ZFP91 overexpression, related to Fig. 3 and Fig. 4.**

**A, B** Gene sets commonly activated (A) or suppressed (B) by ZFP91 knockout and EVI1 knockout in K562 cells, as determined by RNA-seq. **C–E** EVI1 and ZFP91 mRNA (C, D) and protein (E) expression in a ZFP91 deficient HEL clone (HEL-KO3) following rescue with ZFP91 construct carrying cysteine-to-alanine (C-to-A) mutations in the indicated zinc finger (ZnF) domains. “Mock” denotes empty vector control, and “WT” denotes rescue with ZFP91 carrying only a silent mutation conferring resistance to sgZFP91#3.
All bar graphs depict geometric means ± geometric standard deviations. Statistical significance was determined by Welch’s analysis of variance (ANOVA) followed by Dunnett’s test for *EVI1* log-transformed expression (C), or by paired *t* test for *ZFP91* log-transformed expression with adjusted p-values calculated using the Holm-Bonferroni method, with *p<0.05, **p<0.01, ***p<0.001, ****p<0.0001.

**Supplementary Fig. 3: Complementary analyses related to ZFP91 and EVI1, including ATAC-seq footprinting, cell proliferation, expression correlation in AML patient samples, and ZFP91 ChIP-seq at the EVI1 promoter in hepatocellular carcinoma cells, related to Fig. 5 and Discussion.**

**A** Results of footprinting analysis using TOBIAS on ATAC-seq with the JASPAR2024 CORE PFMs motif database. **B** Venn diagram showing the number of less-occupied transcription factors in ZFP91-knockout K562 cells using the two motif databases. **C** Proliferation of EVI1-knockout K562 cells compared to the control. **D, E** A scatter plot of EVI1 and ZFP91 expression in AML patient samples from two public databases. No significant correlation was observed. **F** Expanded view of the *EVI1* promoter regions showing ZFP91 ChIP-seq in hepatocellular carcinoma cell line HepG2. The vertical axis represents fold enrichment of read counts relative to input per bin.

**Supplementary Table 1. A list of oligos and primers, related to Methods and Supplementary Methods.**

Oligos and primers used for this study.

**Supplementary Table 2. A summary of the CRISPR screening, related to Fig. 1 and Supplementary Fig. 1.**

Read count and MAGeCK test results data for all tested genes from the CRISPR screening.
