# Supplementary material for: Genome-wide screening identifies ZFP91 as a key regulator of EVI1 in myeloid leukemia
Source: Oncogene. 2026 Apr 25;45(23):2237–48. doi: 10.1038/s41388-026-03727-7 (PMC13246443; doi:10.1038/s41388-026-03727-7)

Supplementary Figure 1

A

Genes enriched in GFP^{Low}

id	num	score	p-value	fdr	rank	goodsgrna	lfc
HEATR1	6	1.81E-12	2.28E-07	0.00055	1	6	1.1296
MYC	6	3.24E-12	2.28E-07	0.00055	2	6	1.9367
RFK	6	2.91E-10	2.28E-07	0.00055	3	4	1.0361
MECOM	6	1.55E-09	2.28E-07	0.00055	4	6	0.9259
ZFP91	6	3.04E-09	2.28E-07	0.00055	5	6	0.9828
SLC52A2	6	3.97E-09	2.28E-07	0.00055	6	6	1.0249
MYB	6	6.90E-09	2.28E-07	0.00055	7	6	1.2951
DYRK1A	6	5.06E-08	2.28E-07	0.00055	8	6	0.9505
GAB2	6	3.22E-07	2.28E-07	0.00055	9	5	0.7874
PES1	6	1.57E-06	1.60E-06	0.00347	10	5	0.8102
CBFA2T3	6	2.15E-06	2.05E-06	0.00405	11	5	0.8366
GATA1	6	3.39E-06	2.96E-06	0.00536	12	5	0.7773
AMBRA1	6	3.55E-06	3.42E-06	0.00571	13	5	0.8100
SEPHS1	6	6.32E-06	1.07E-05	0.01662	14	5	0.4075
ZEB2	6	6.85E-06	1.21E-05	0.01749	15	6	1.0693
SLC7A1	6	1.05E-05	1.98E-05	0.02692	16	5	0.8473
SEC61A1	6	1.23E-05	2.71E-05	0.03465	17	5	0.8278
MRPS14	6	1.74E-05	4.63E-05	0.05583	18	4	0.8005
RREB1	6	2.21E-05	7.00E-05	0.07599	19	3	0.4561
TSACC	6	2.48E-05	7.37E-05	0.07614	20	2	-0.2352
ZNRD1	6	3.07E-05	8.69E-05	0.08244	21	6	0.6789
C1orf185	6	3.14E-05	8.73E-05	0.08244	22	4	0.8317
hsa-mir-1205	4	4.16E-05	6.95E-05	0.07599	23	4	0.8620
PPRC1	6	4.50E-05	1.54E-04	0.13923	24	6	0.6313
RPS18	6	5.35E-05	1.74E-04	0.14642	25	6	0.4560
CEP97	6	5.74E-05	1.85E-04	0.14870	26	5	0.7281
UTP18	6	5.99E-05	1.96E-04	0.14971	27	6	0.5561
DCAF8	6	6.38E-05	2.00E-04	0.14971	28	6	0.4986
INPPL1	6	6.47E-05	2.11E-04	0.15047	29	5	0.7747
OR5H14	6	6.57E-05	2.15E-04	0.15047	30	3	0.2153
WDR43	6	7.04E-05	2.25E-04	0.15047	31	5	0.4946
TPGS2	6	7.45E-05	2.29E-04	0.15047	32	3	0.3123
hsa-mir-34c	4	9.44E-05	1.75E-04	0.14642	33	3	0.6352
GRB2	6	9.78E-05	2.99E-04	0.18661	34	3	0.1977
NUPL1	6	1.00E-04	3.04E-04	0.18661	35	6	0.4907
TMEM132B	6	1.06E-04	3.09E-04	0.18661	36	4	0.5371
ANKRD34B	6	1.20E-04	3.43E-04	0.19573	37	5	0.3832
TNFSF13	6	1.25E-04	3.61E-04	0.19573	38	3	0.0843
CKS1B	6	1.26E-04	3.64E-04	0.19573	39	6	0.3028
MED19	6	1.28E-04	3.67E-04	0.19573	40	6	0.7216
S100A7A	6	1.29E-04	3.70E-04	0.19573	41	6	0.3755
FBXL16	6	1.40E-04	4.23E-04	0.21266	42	6	0.3817
RNLS	6	1.45E-04	4.50E-04	0.21266	43	6	0.2666
KLF1	6	1.49E-04	4.57E-04	0.21266	44	5	0.6200
IL18RAP	6	1.49E-04	4.57E-04	0.21266	45	4	0.1325
MBTPS1	6	1.52E-04	4.60E-04	0.21266	46	6	0.3463
C1QTNF4	6	1.75E-04	5.36E-04	0.24227	47	4	0.7725

B

Genes enriched in GFP^{High}

id	num	score	p-value	fdr	rank	goodsgrna	lfc
IARS2	6	3.89E-10	2.28E-07	0.00248	1	6	-1.75200
STAG2	6	4.79E-09	2.28E-07	0.00248	2	6	-0.79559
LETM1	6	1.46E-06	6.84E-07	0.00495	3	5	-0.79327
SMU1	6	2.43E-06	1.14E-06	0.00619	4	6	-0.86180
TSC2	6	5.66E-06	4.33E-06	0.01881	5	5	-0.53786
OR4F5	4	1.91E-05	1.16E-05	0.04208	6	3	-0.81356
USP17L11	6	2.27E-05	3.90E-05	0.10066	7	5	-0.16445
URB2	6	2.48E-05	4.13E-05	0.10066	8	2	-0.03609
CCNA2	6	2.50E-05	4.17E-05	0.10066	9	5	-0.80770
DEPDC5	6	3.38E-05	6.23E-05	0.11910	10	5	-0.64502
SPDYE1	6	3.52E-05	6.27E-05	0.11910	11	2	0.02720
DDIT4	6	3.93E-05	6.59E-05	0.11910	12	6	-0.51364
ANO7	6	4.49E-05	7.87E-05	0.11910	13	3	-0.45675
ARL4A	6	4.94E-05	9.05E-05	0.11910	14	4	-0.65652
OR2L3	6	5.23E-05	9.19E-05	0.11910	15	5	-0.59569
CIT	6	5.35E-05	9.33E-05	0.11910	16	4	-0.66269
CBX8	6	5.35E-05	9.33E-05	0.11910	17	6	-0.53488
RAD17	6	7.45E-05	1.38E-04	0.16584	18	2	0.11728
TICRR	6	1.02E-04	1.95E-04	0.21114	19	4	-0.51855
BAG2	6	1.18E-04	2.28E-04	0.21214	20	5	-0.52018
IGFBP1	6	1.19E-04	2.28E-04	0.21214	21	4	-0.39871
ATP6V0C	6	1.24E-04	2.31E-04	0.21214	22	3	-0.09996
SPATA20	6	1.28E-04	2.37E-04	0.21214	23	5	-0.48134
SLC25A15	6	1.34E-04	2.53E-04	0.21214	24	3	-0.14676
PSMA5	6	1.36E-04	2.55E-04	0.21214	25	5	-0.52257
LRRC18	6	1.40E-04	2.64E-04	0.21214	26	4	-0.63959
CLN6	6	1.53E-04	3.03E-04	0.23097	27	3	-0.15056
NR2F2	6	1.68E-04	3.23E-04	0.23097	28	5	-0.70740
HSPE1-MOB4	6	1.74E-04	3.34E-04	0.23097	29	2	0.05961
FAM69B	6	1.77E-04	3.34E-04	0.23097	30	4	-0.28171
C7orf61	6	1.81E-04	3.40E-04	0.23097	31	6	-0.49510
DPH6	3	1.86E-04	1.61E-04	0.18369	32	1	-0.10865

C

Genes enriched in GFP^{High}

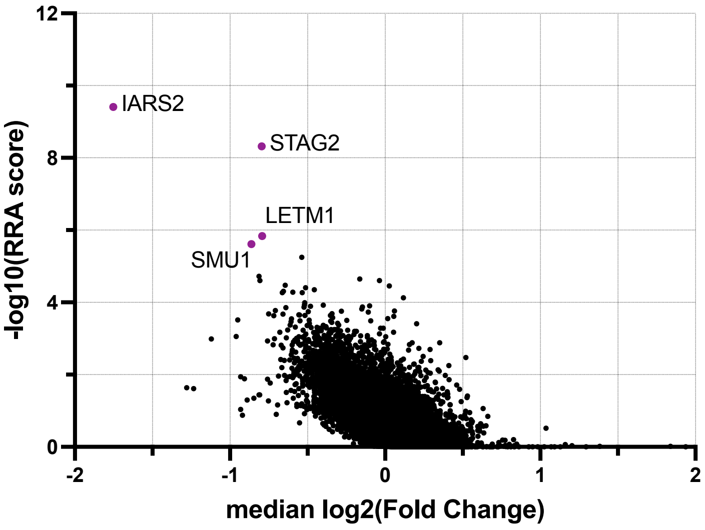

D

Validation in K562-EVI1-GFP-Cas9 cells

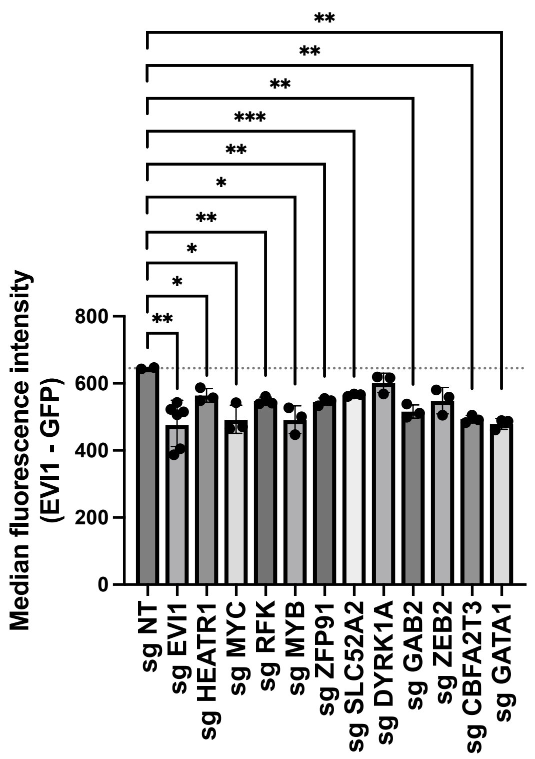

E

ZFP91 overexpression

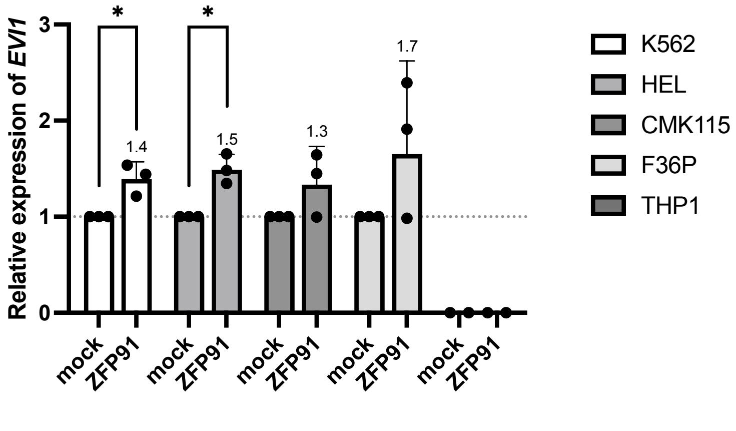

F

ZFP91 overexpression

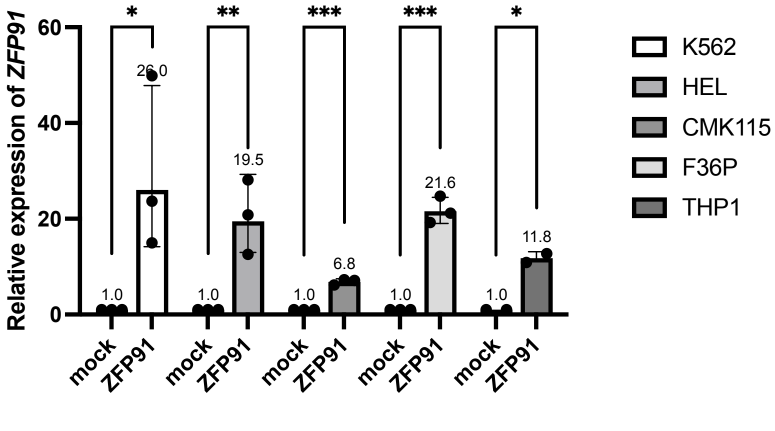

Supplement: Supplementary file 2 — Supplementary Figure 1 [file 41388_2026_3727_MOESM2_ESM.pdf]
