# Supplementary figures and images for: Genome-wide screening identifies ZFP91 as a key regulator of EVI1 in myeloid leukemia
Source: Oncogene. 2026 Apr 25;45(23):2237–48. doi: 10.1038/s41388-026-03727-7 (PMC13246443; doi:10.1038/s41388-026-03727-7)

Supplementary Figure 3

A

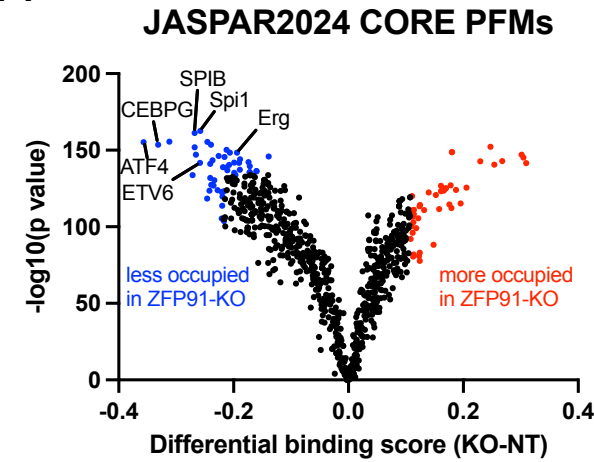

B

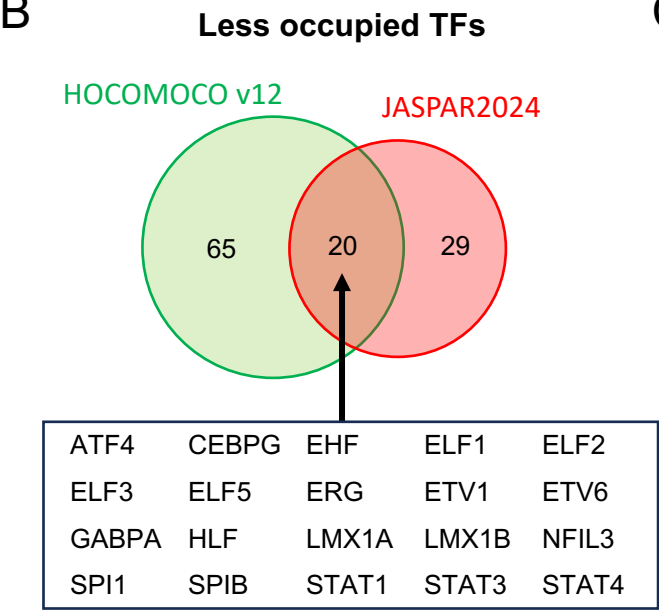

C

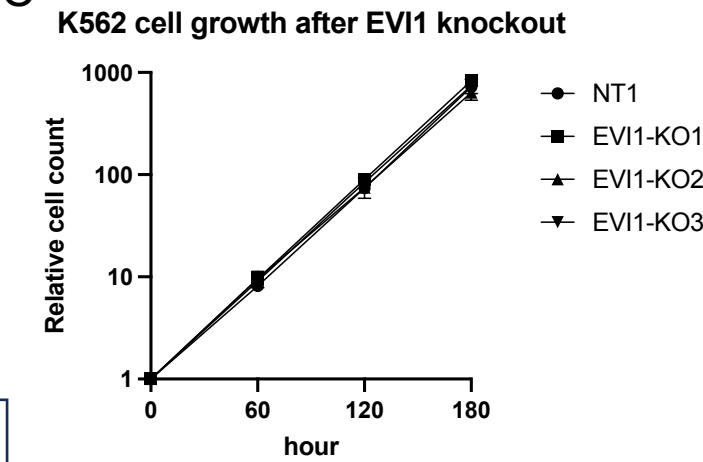

D

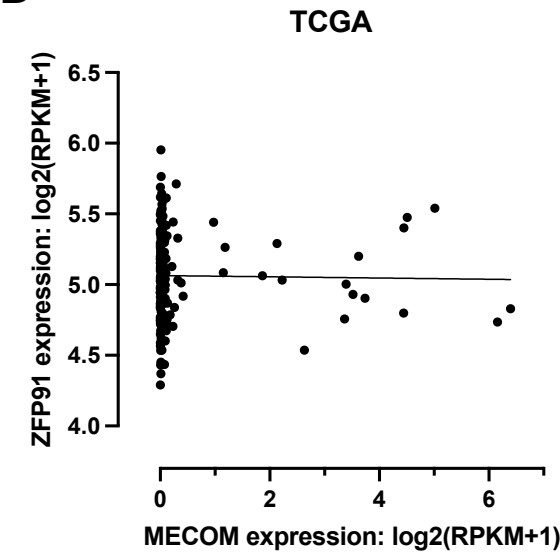

E

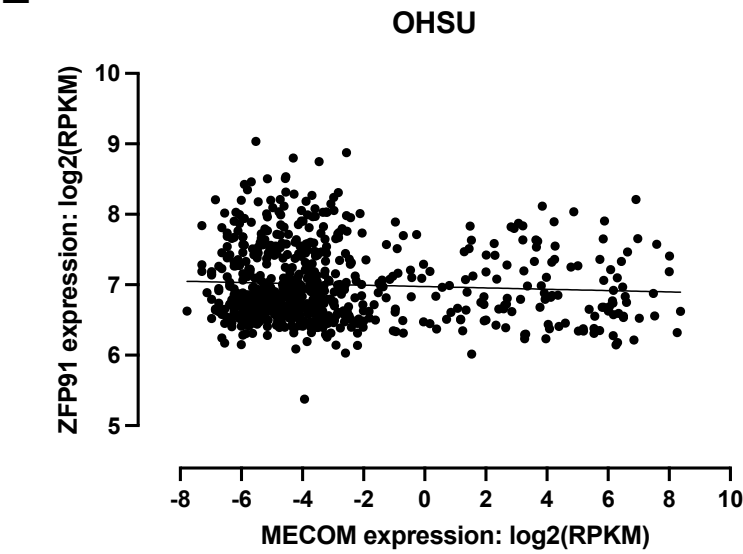

F

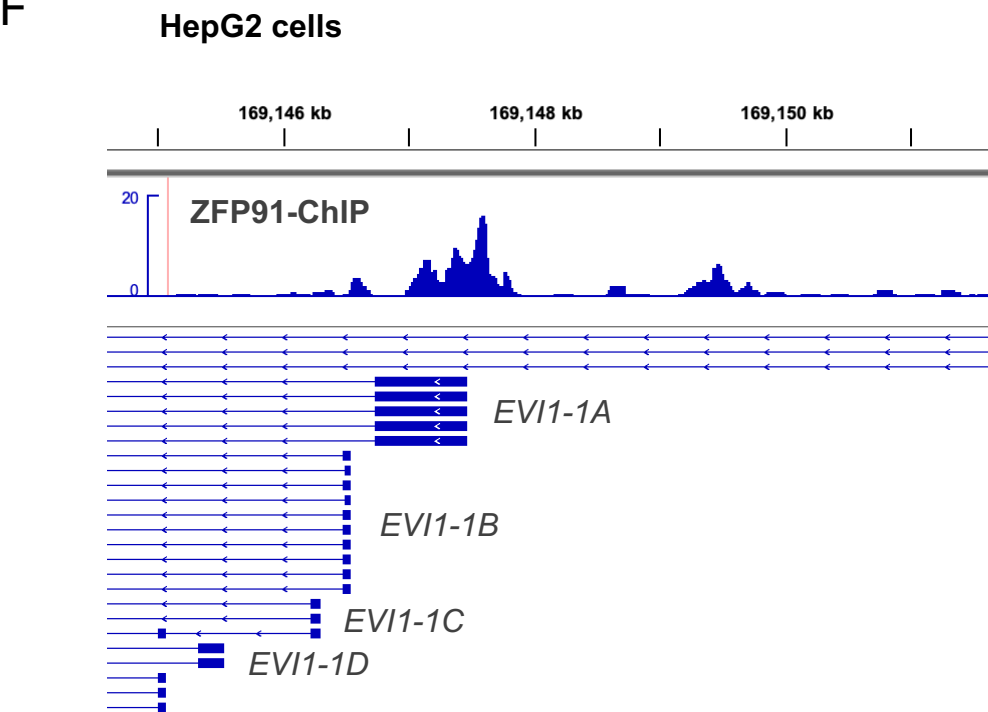

Supplement: Supplementary file 4 — Supplementary Figure 3 [file 41388_2026_3727_MOESM4_ESM.pdf]
