# Supplementary material for: Genome-wide screening identifies ZFP91 as a key regulator of EVI1 in myeloid leukemia
Source: Oncogene. 2026 Apr 25;45(23):2237–48. doi: 10.1038/s41388-026-03727-7 (PMC13246443; doi:10.1038/s41388-026-03727-7)

Supplementary Figure 2

A

ONTOLOGY	ID	Description	setSize	ZFP91-KO vs. control NES	ZFP91-KO vs. control p.adjust	ZFP91-KO vs. control qvalue	EVI1-KO vs. control NES	EVI1-KO vs. control p.adjust	EVI1-KO vs. control qvalue
CC	GO:0030312	external encapsulating structure	221	1.69	3.74E-03	3.62E-03	1.70	1.04E-03	9.41E-04
CC	GO:0062023	collagen-containing extracellular matrix	180	1.69	1.54E-02	1.50E-02	1.64	8.75E-03	7.89E-03
CC	GO:0031012	extracellular matrix	220	1.67	3.73E-03	3.61E-03	1.70	1.04E-03	9.41E-04

B

ONTOLOGY	ID	Description	setSize	ZFP91-KO vs. control NES	ZFP91-KO vs. control p.adjust	ZFP91-KO vs. control qvalue	EVI1-KO vs. control NES	EVI1-KO vs. control p.adjust	EVI1-KO vs. control qvalue
BP	GO:0140053	mitochondrial gene expression	164	-2.28	8.28E-07	8.03E-07	-2.50	1.31E-11	1.18E-11
BP	GO:0032543	mitochondrial translation	130	-2.27	5.41E-06	5.25E-06	-2.43	1.80E-08	1.62E-08
BP	GO:0022613	ribonucleoprotein complex biogenesis	460	-1.61	1.19E-04	1.15E-04	-2.85	1.91E-38	1.72E-38
BP	GO:0006364	rRNA processing	222	-1.71	4.62E-03	4.48E-03	-2.95	2.55E-26	2.30E-26
BP	GO:0042254	ribosome biogenesis	316	-1.61	4.63E-03	4.49E-03	-2.98	3.45E-33	3.11E-33
BP	GO:0034470	ncRNA processing	410	-1.48	1.86E-02	1.80E-02	-2.79	2.01E-32	1.81E-32
BP	GO:0016072	rRNA metabolic process	259	-1.60	3.24E-02	3.14E-02	-2.88	6.05E-26	5.45E-26
CC	GO:0098798	mitochondrial protein-containing complex	289	-1.94	1.61E-06	1.56E-06	-2.17	3.26E-11	2.94E-11
CC	GO:0000313	organellar ribosome	89	-2.13	6.52E-04	6.32E-04	-2.46	1.33E-07	1.20E-07
CC	GO:0005761	mitochondrial ribosome	89	-2.13	6.52E-04	6.32E-04	-2.46	1.33E-07	1.20E-07
CC	GO:0005743	mitochondrial inner membrane	452	-1.50	2.19E-03	2.12E-03	-1.61	2.36E-05	2.12E-05
CC	GO:0019866	organelle inner membrane	495	-1.47	3.54E-03	3.43E-03	-1.56	7.38E-05	6.65E-05
CC	GO:0005840	ribosome	226	-1.63	1.59E-02	1.54E-02	-2.09	2.15E-08	1.94E-08
CC	GO:0000315	organellar large ribosomal subunit	56	-2.00	4.18E-02	4.06E-02	-2.28	4.11E-04	3.70E-04
CC	GO:0005762	mitochondrial large ribosomal subunit	56	-2.00	4.18E-02	4.06E-02	-2.28	4.11E-04	3.70E-04

C

ZnF-mutant ZFP91 rescue in ZFP91-KO HEL cells

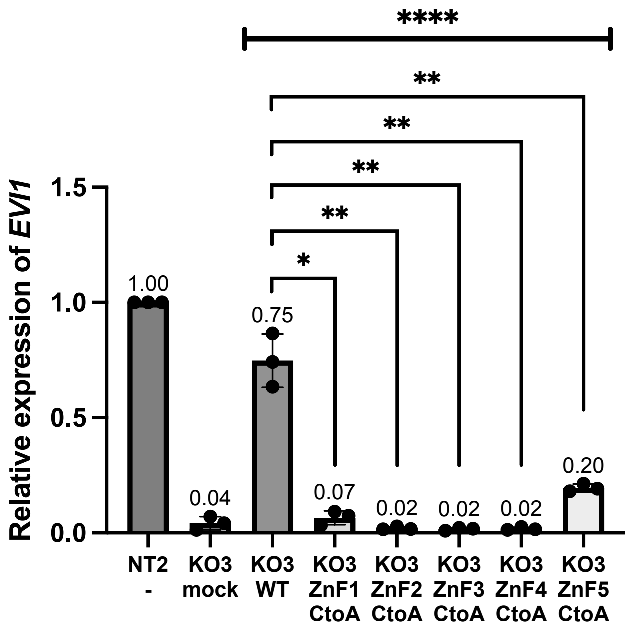

D

ZnF-mutant ZFP91 rescue in ZFP91-KO HEL cells

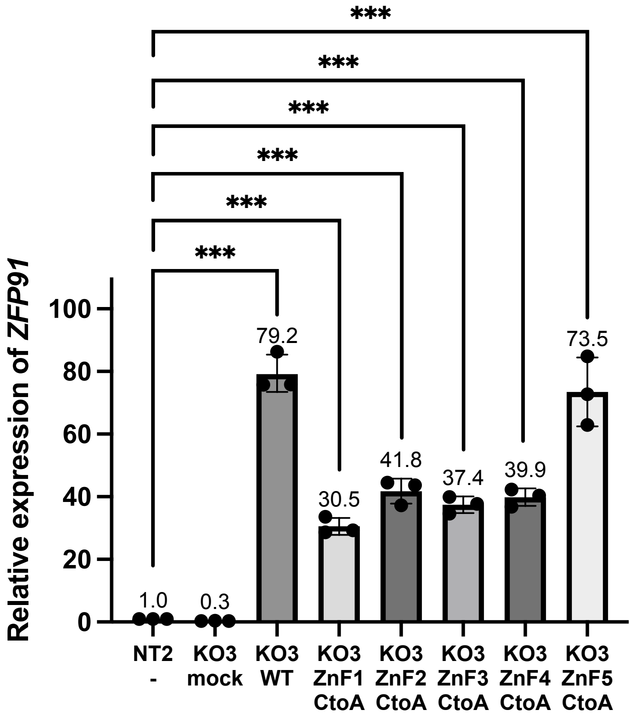

E

HEL cells

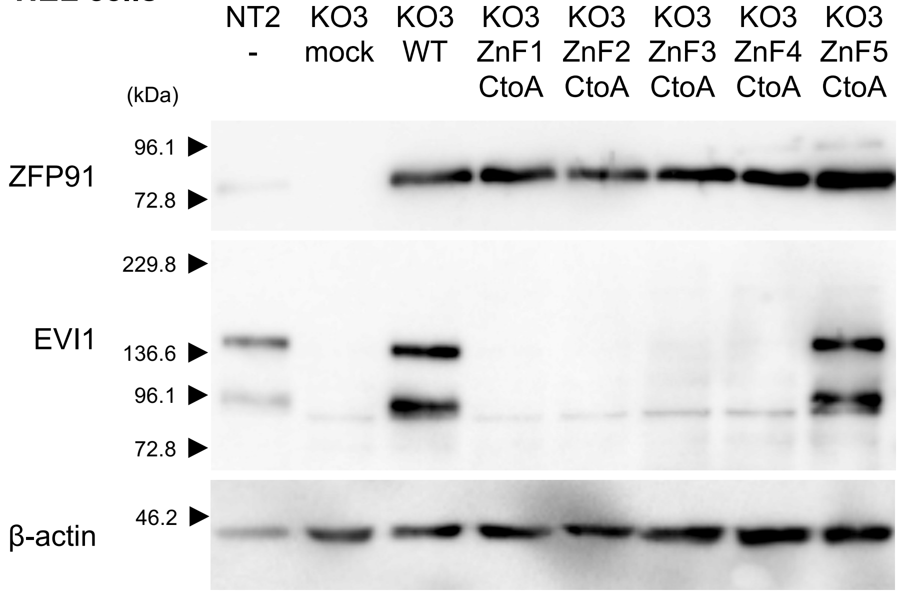

Supplement: Supplementary file 3 — Supplementary Figure 2 [file 41388_2026_3727_MOESM3_ESM.pdf]
